# Stable isotopes unveil one millennium of domestic cat paleoecology in Europe

**DOI:** 10.1038/s41598-022-16969-8

**Published:** 2022-07-27

**Authors:** Magdalena Krajcarz, Wim Van Neer, Maciej T. Krajcarz, Danijela Popović, Mateusz Baca, Bea De Cupere, Quentin Goffette, Hans Christian Küchelmann, Anna Gręzak, Urszula Iwaszczuk, Claudio Ottoni, Katrien Van de Vijver, Jarosław Wilczyński, Anna Mulczyk, Jan Wiejacki, Daniel Makowiecki, Hervé Bocherens

**Affiliations:** 1 Institute of Archaeology, Nicolaus Copernicus University in Toruń, Szosa Bydgoska 44/48, 87-100 Toruń, Poland; 2 Operational Directorate Earth and History of Life, Royal Belgian Institute of Natural Sciences, Vautierstraat 29, 1000 Brussels, Belgium; 3 Laboratory of Biodiversity and Evolutionary Genomics, University of Leuven, Charles Deberiotstraat 32, 3000 Leuven, Belgium; 4 Research Centre in Warszawa, Institute of Geological Sciences, Polish Academy of Sciences, Twarda 51/55, 00-818 Warsaw, Poland; 5 Centre of New Technologies, University of Warsaw, S. Banacha 2c, 02-097 Warsaw, Poland; 6 Knochenarbeit, Speicherhof 4, 28217 Bremen, Germany; 7 Department of Bioarchaeology, Faculty of Archaeology, University of Warsaw, Krakowskie Przedmieście 26/28, 00-927 Warsaw, Poland; 8 Institute of Mediterranean and Oriental Cultures, Polish Academy of Sciences, Nowy Świat 72, 00-330 Warsaw, Poland; 9 Centre of Molecular Anthropology for Ancient DNA Studies, Department of Biology, University of Rome “Tor Vergata”, Via della Ricerca Scientifica 1, 00133 Rome, Italy; 10 Institute of Systematics and Evolution of Animals, Polish Academy of Sciences, Sławkowska 17, 31-016 Kraków, Poland; 11 Senckenberg Centre for Human Evolution and Palaeoenvironment, University of Tübingen, Sigwartstrasse 10, 72076 Tübingen, Germany; 12 Biogeology, Department of Geosciences, University of Tübingen, Hölderlinstrasse 12, 72074 Tübingen, Germany

**Keywords:** Palaeoecology, Stable isotope analysis

## Abstract

The domestic cat is the world's most popular pet and one of the most detrimental predators in terrestrial ecosystems. Effective protection of wildlife biodiversity demands detailed tracking of cat trophic ecology, and stable isotopes serve as a powerful proxy in dietary studies. However, a variable diet can make an isotopic pattern unreadable in opportunistic predators. To evaluate the usefulness of the isotopic method in cat ecology, we measured C and N isotope ratios in hundreds of archaeological cat bones. We determined trends in cat trophic paleoecology in northern Europe by exploiting population-scale patterns in animals from diverse locations. Our dataset shows a high variability of isotopic signals related to the socio-economic and/or geomorphological context. This points toward regularities in isotopic patterns across past cat populations. We provide a generalized guide to interpret the isotopic ecology of cats, emphasizing that regional isotopic baselines have a major impact on the isotopic signal.

## Introduction

### Cat ecology

From an ecological perspective, the domestic cat is a very flexible animal. Despite being a hypercarnivore (i.e., highly dependent on a daily supply of meat), cats select prey opportunistically^1,2^. They can adjust to various conditions and successfully colonize a broad spectrum of environments^1,3^. Even when fed enough by their human owners, they search for other food sources and easily become feral (fully independent of people)^1,3^.

The history of the domestic cat and its ecological adaptations are currently highly debated topics^4^. Its domestication and rodent-controlling ability, followed by its global dispersal, is essential for our understanding of the diversity and evolution of human–animal relationships and the history of husbandry^5–10^. The domestic cat has become a widespread and abundant predator, and is considered one of the most detrimental species in the world that negatively impacts a wide range of wild native species^3,11–14^. Domestic cats also pose a serious threat to wildcat native populations through hybridization^15,16^ and competition^17,18^. Thus, the study of domestic cat ecology is important from various points of view—from the history of one of the world's most popular pet to conservation biology.

### History of the domestic cat and its ecology

The earliest history of the human–cat relationship is still poorly documented, due to the sporadic occurrence of cat bones in archaeological and paleontological contexts, with records from the early stages of human–cat relationships containing long time gaps^7–10,19,20^. The Near Eastern ancestor of domestic cats had already emerged in Central Europe 6000 years ago; however, the possible relationships of this early population with modern domestic cats are unknown^7,8,10^. Still, the most plausible hypothesis assumes that the Roman Empire, with its military campaigns and far-reaching trade, played a major role in establishing a permanent domestic cat population across Europe^7,21,22^. However, archaeological evidence indicates that cats only became a constant and frequent component of the European anthropic landscape during the Middle Ages^21,22^. Viking expansion, followed later by trade contacts with the Ottoman Empire, played an important role in further distributing cats across the continent, and establishing the modern-day population^7,19,21,23^. During this period, cats became important pest controllers, and their numbers increased in parallel to the expansion of towns^22^. The high concentration of mice and rats and easy access to human food waste allowed dense cat populations to develop^1,24^.

### Stable isotopes in tracking past cat ecology

The possibility of using stable isotopes to reconstruct diet became a powerful tool in archaeological studies focused on elucidating past human and animal ways of life^25–27^. For decades, this method has been used to detect past diets, providing insights into animal trophic ecology and adaptation to the anthropogenic landscape^28,29^.

The domestic cat is a complex object for stable isotope analysis because it opportunistically selects a wide range of food sources^1,2^. However, because it is a hypercarnivore, the bulk of potential food sources is relatively well defined. Until now, only few studies have investigated the ancient or modern cat diets using stable isotopes^10,30–34^. Here, we aimed to investigate the pattern of carbon and nitrogen isotopic variability in domestic cats from Northern Europe. Through analyzing a vast database of bulk collagen δ^13^C and δ^15^N values and comparing environmental and cultural conditions, we explore: (i) the range of isotopic variability among cats; (ii) predictability of cat isotopic signal in the context of its environment and presumed ecology; and (iii) usefulness of the stable isotope method to monitor the ecology and diet of this opportunistic and individualistic carnivore.

We used a collection of archaeological cat bones from medieval and post-medieval Europe, ranging from the 6th to the eighteenth centuries AD (Datasets S1 and S2). Due to the very low number of finds, which hindered statistical representativeness, we did not retain the earliest available cats (from Neolithic to Antiquity). We also excluded animals from the 19th to twenty-first century populations (i.e., cats from industrial times), as manufactured food and artificial pollution potentially strongly influenced their isotopic signal, making it difficult to interpret^35,36^. Our material originated from northern Europe, which is a region large enough to represent variable landscapes and socio-economic contexts, but is sufficiently restricted to avoid covering extremely different ecosystems, which could dominate the isotopic ecology and obscure minute differences.

Cat remains are scarce at archaeological sites. Complete or partial carcasses occur sometimes, but usually only single bones are found. Moreover, these finds often occur in archaeological contexts that provide limited information on cat ecology and relationship with people (such as latrines and waste heaps, or hideouts and natural shelters located far from human settlements). Therefore, our knowledge on the behavior, habitat, and place in the human cultural landscape of ancient cats is still limited. Thus, rather than deliberate over individual isotopic traits, we exploited isotopic trends among broad groups of cats in relation to their chronology, geography, and socio-economic contexts to determine population-scale isotopic patterns.

## Results

### Stable isotope results

**Figure 1:**
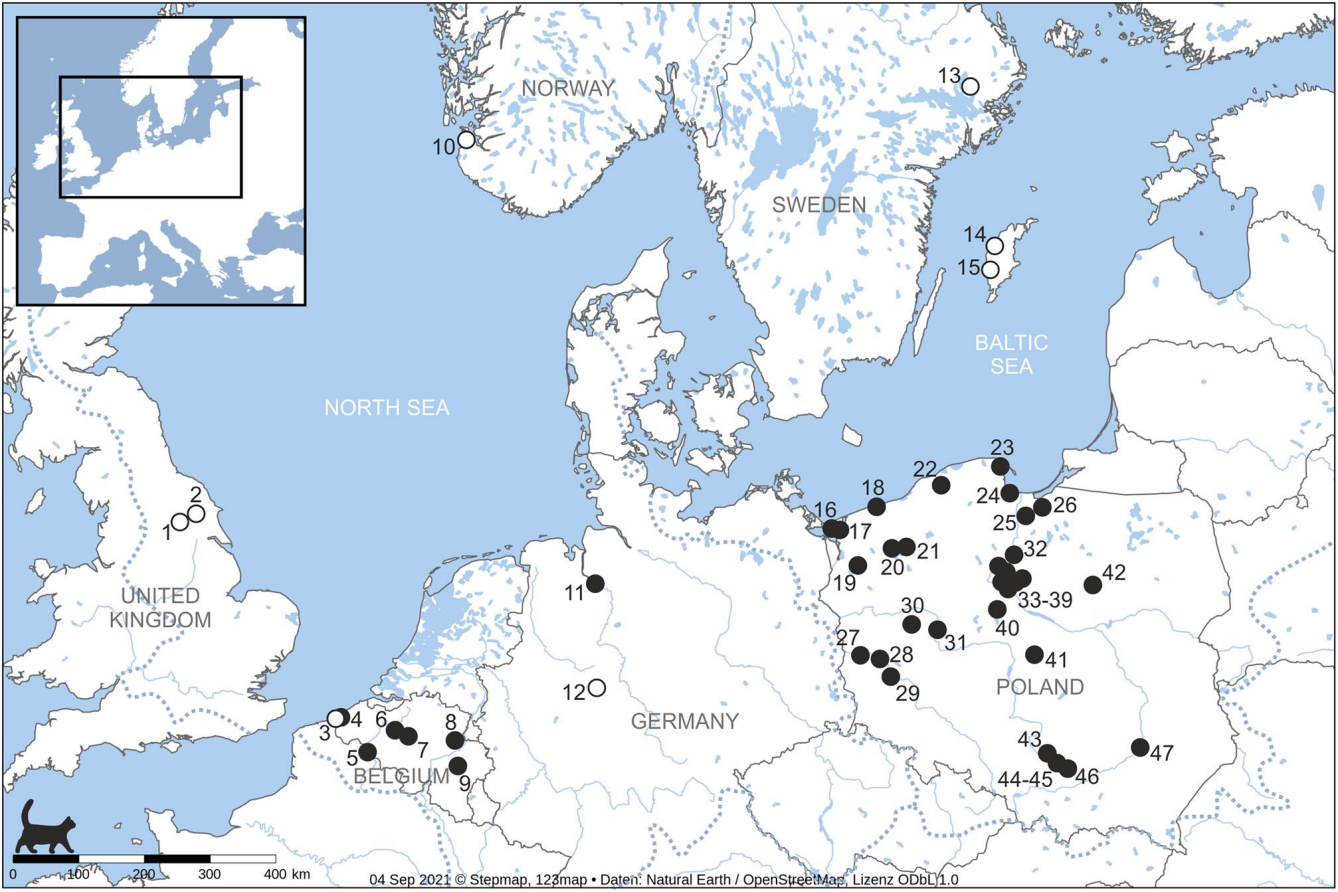
Locations with medieval and post-medieval cat remains studied for stable isotopes (1—York, 2—Wharram, 3—Koksijde, 4—Nieuwpoort, 5—Tournai, 6—Brussel, 7—Aalst, 8—Tongeren, 9—Logne, 10—Stavanger, 11—Bremen, 12—Dalheim, 13—Sigtuna, 14—Visby, 15—Ridanäs, 16—Lubin, 17—Wolin, 18—Kołobrzeg, 19—Stargard, 20—Żółte, 21—Stare Drawsko, 22—Słupsk, 23—Puck, 24—Gdańsk, 25—Malbork, 26—Elbląg, 27—Krosno Odrzańskie, 28—Zawada, 29—Bytom Odrzański, 30—Chełmno, 31—Poznań, 32—Grudziądz, 33—Kałdus, 34—Starogród, 35—Papowo Biskupie, 36—Zamek Bierzgłowski, 37—Toruń, 38—Gronowo, 39—Napole, 40—Karczyn, 41—Tum, 42—Grudusk, 43—Deszczowa Cave, 44—Perspektywiczna Cave, 45—Shelter in Udorka Valley I, 46—Miechów, 47—Sandomierz). Black circles—locations with new data; open circles—locations with literature data (see “Classification of the sites” section). Dotted lines show contours of the North Sea and Baltic Sea catchment basins.

**Figure 2:**
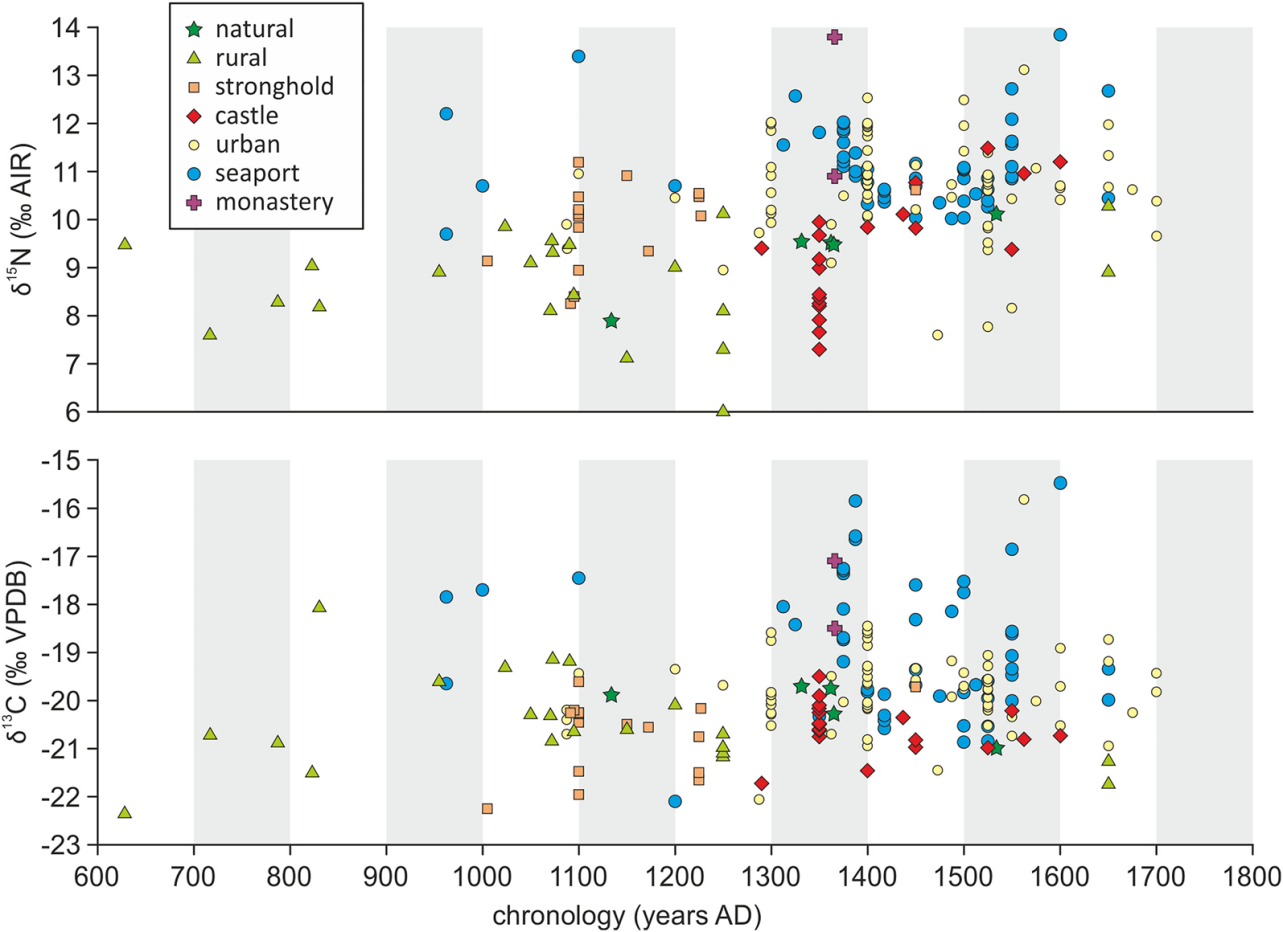
δ^13^C and δ^15^N values vs. specimen chronology in the cats analyzed in this study. The chronology reported for each specimen is the central point (± 1-year accuracy) of the site chronology range or the central point of the 95.4% probability range of the radiocarbon date. For full chronology ranges see Datasets S1 and S2 and for each socio-economic context shown separately see SI Appendix, Figs. S1 and S2.

We obtained new reliable isotopic results for n = 170 domestic cat individuals from 58 archaeological sites at 39 locations from Poland, Belgium, and Germany (Figs. 1 and 2; Datasets S1 and S2). Collagen quality checks (based on C:N atomic ratios) ranged from 2.9 to 3.6 (most were within 3.2–3.4), which was within the acceptable range for uncontaminated and non-degraded collagen^37^ (Dataset S2). Together with literature data taken from previously published studies on 8 additional locations^38–45^, we analyzed 188 individuals in total (Dataset S1). The isotopic values of domestic cats ranged from 6.0 to 13.8‰ for δ^15^N and from − 22.4 to − 15.5‰ for δ^13^C (Dataset S1).

### Cat isotope signal along the timeline

The studied dataset included cat remains dating between AD ~ 600 and AD 1800 (Fig. 2; Datasets S1 and S2). Precise temporal fluctuations in the isotopic signal were difficult to detect. Many samples had wide chronological ranges (covering up to 550 years in some specimens, due to the lack of diagnostic archaeological artifacts or weak stratigraphic dating of some sites). However, a distinct shift in ranges of both δ^13^C and δ^15^N values appeared around the thirteenth century. This finding was supported by statistical analysis, which revealed significant differences in the variance of pre-AD 1250 and post-AD 1250 samples (Kruskal–Wallis test's *p* = 0.0001405 for δ^13^C and *p* < 10^–7^ for δ^15^N values) (SI Appendix Tables S2 and S3). This change included an increase in mean values (from − 20.3 to − 19.6‰ for δ^13^C, and from 9.5 to 10.6‰ for δ^15^N values) (SI Appendix Table S1). However, overall variability did not change (standard deviation from 1.1 to 1.2 for δ^13^C, and 1.2 to 1.3 for δ^15^N values) (SI Appendix Table S1). This shift coincided with the emergence of new types of human settlements, such as towns and castles^46^, and is represented in our database by the prevalence of different site types among the earlier and later periods (Fig. 2). Of note, the pre-AD 1250 cats in our database almost exclusively originated from the eastern part of the studied area (mainly Poland), while the post-AD 1250 samples originated from the entire studied area. Regarding the eastern region only, the statistical differences in δ^13^C and δ^15^N values between the pre- and post-AD 1250 cats were still significant (SI Appendix Tables S2 and S3).

### Cat isotope signal vs. distance from the sea

**Figure 3:**
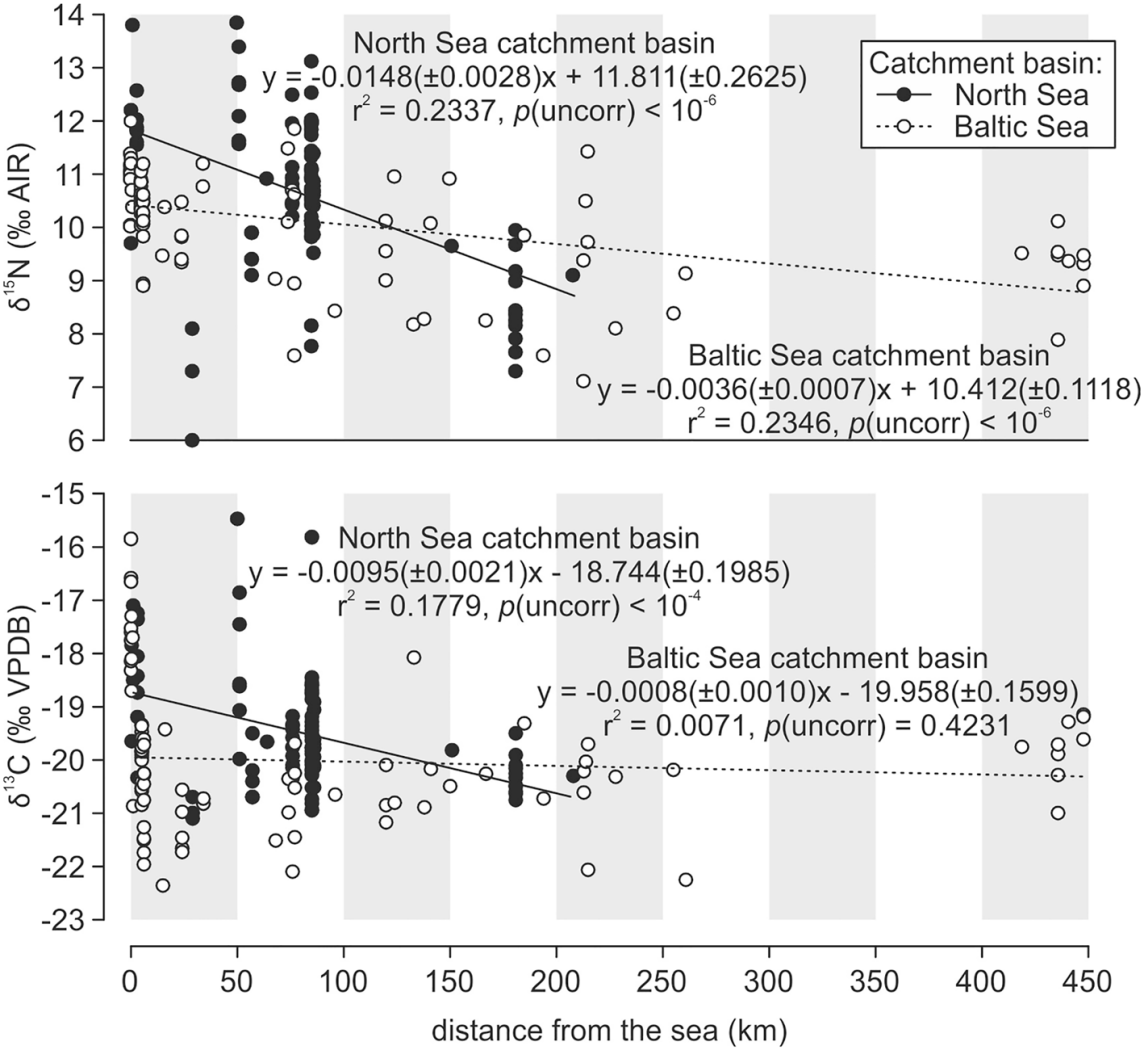
δ^13^C and δ^15^N values in cats vs. site distance from the sea.

We observed a significant correlation between δ^15^N values and distance from the sea for all our samples (Fig. 3, supporting statistics shown in Dataset S8). The δ^15^N values tended to decrease with increasing distance, with the rate of this shift being higher in sites located in the North Sea catchment area (Belgium, Germany, Norway, and UK; on average, 1.49‰ per 100 km) compared to the Baltic Sea region (Poland and Sweden; on average, 0.36‰ per 100 km) (Fig. 3). In the case of δ^13^C values, this correlation was weaker and only visible for cat samples from the North Sea catchment area (Fig. 3).

δ^13^C and δ^15^N values differed in coastal cats from the North Sea and Baltic Sea regions (averages being higher for the North Sea, SI Appendix Tables S4–S7), following an isotope pattern already known for marine fish^47,48^. This was probably connected to general isotopic differences in the nutrient bases of the two basins. Baltic fish tend to have lower δ^13^C and δ^15^N values^47,48^; consequently, the isotopic signal of marine resources in the coastal areas of the Baltic region was less dissimilar to the terrestrial signals of inland resources. This observation supported the subdivision of our dataset into two geographic groups of cats, which were connected to two different marine-derived isotope baselines: cats from the North Sea region and cats from the Baltic Sea region. For this reason, we analyzed the data separately for these two sea catchment areas.

### Cat isotope signal vs. socio-economic context of the site

**Figure 4:**
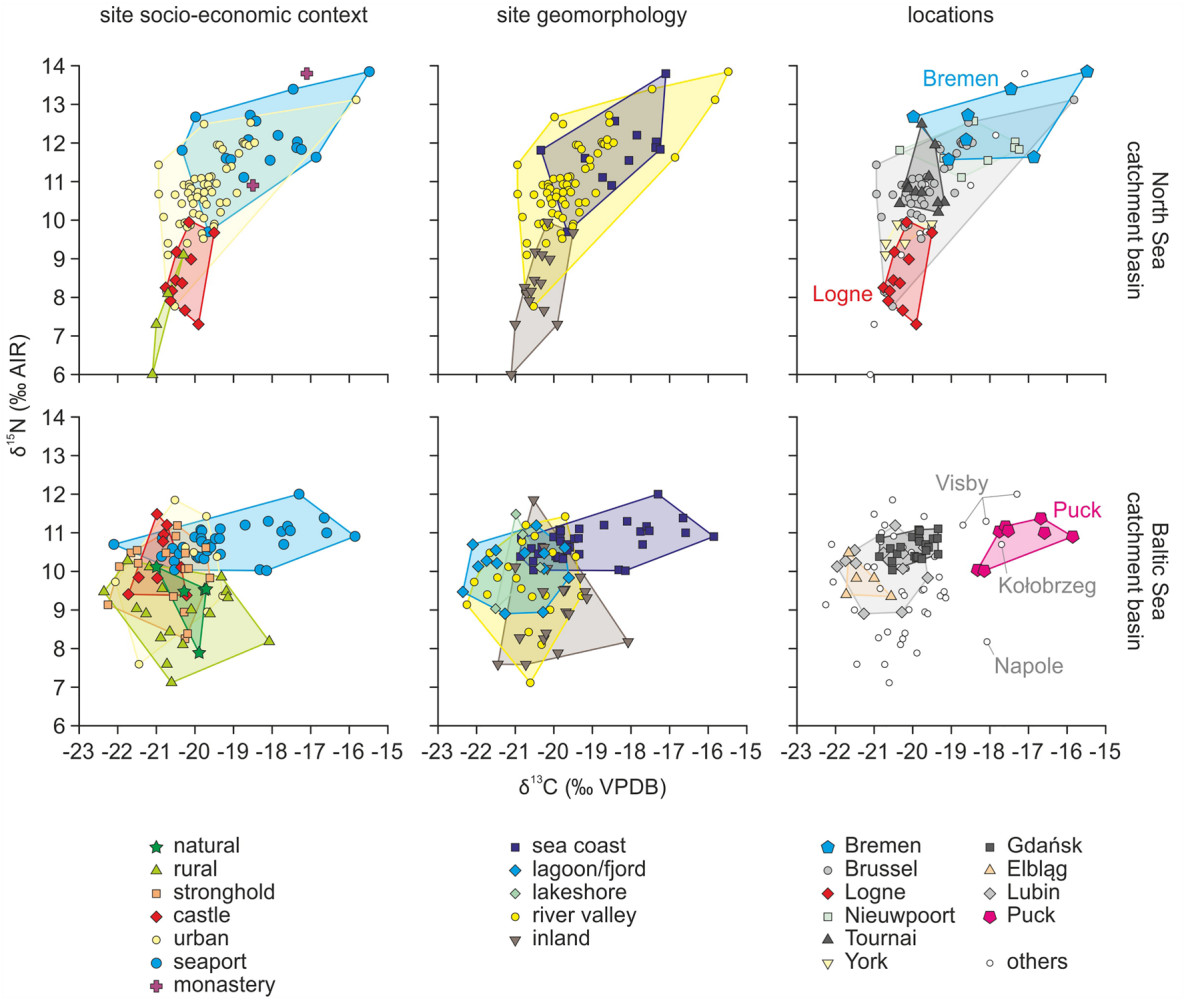
δ^13^C and δ^15^N values in cats vs. site socio-economic context, site geomorphology and locations (divided by sea catchment area). Colored fields are convex hulls covering samples attributed to each group. In locations panels, the locations that yielded < 5 specimens are denoted as ‘others’.

**Figure 5:**
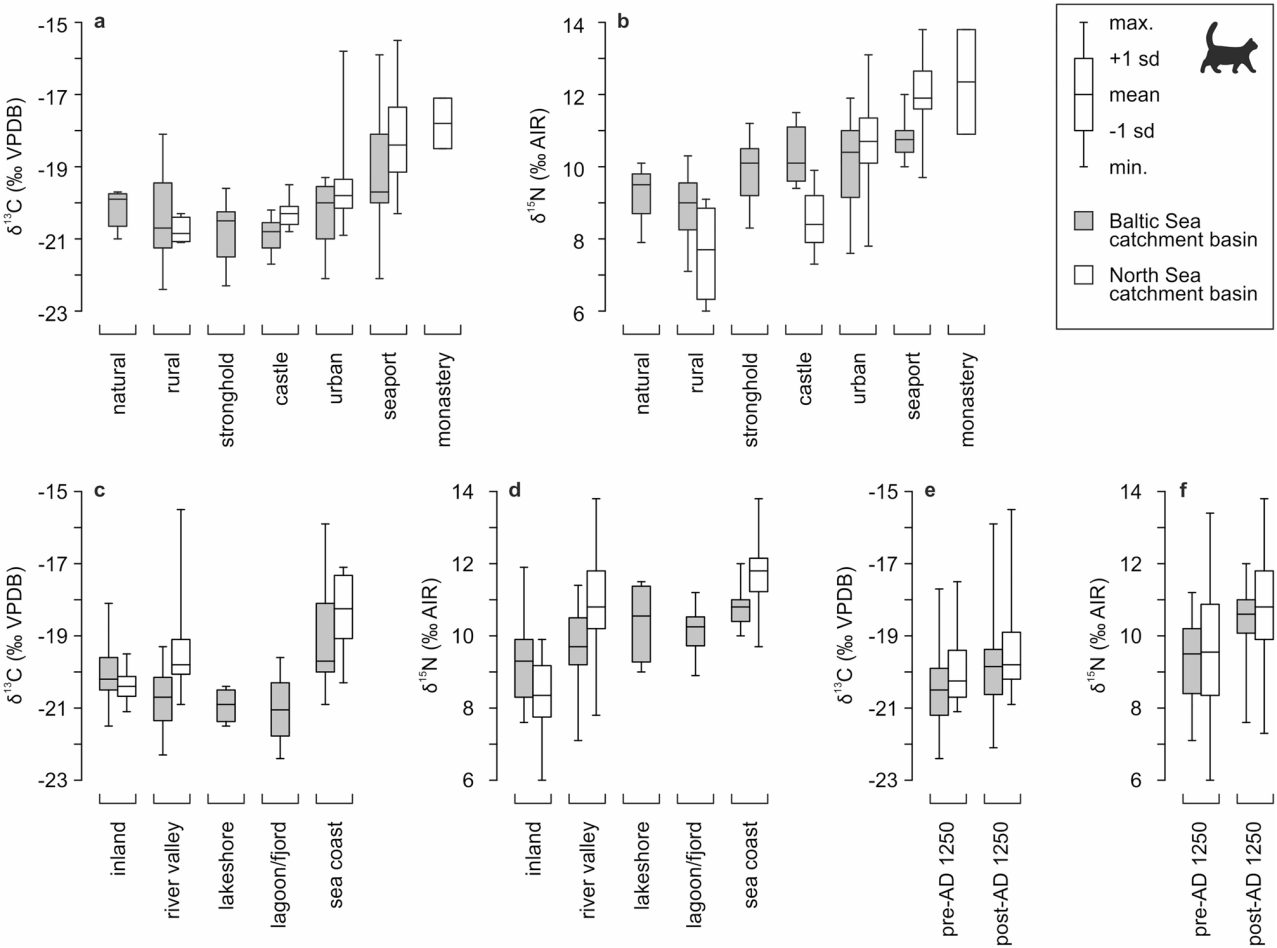
Variability of δ^13^C and δ^15^N values in medieval and post-medieval cats grouped by: (**a**,**b**) site socio-economic context; (**c**,**d**) site geomorphology; (**e**,**f**) pre- and post-AD 1250 chronology. For detailed values see SI Appendix Tables S1, S4–S7.

Among the sites located in the Baltic region, cats from strongholds, castles, rural, natural and urban sites were statistically similar to each other in terms of δ^13^C values (analysis of variance *p* > 0.1 for each of these pairs for the null hypothesis of equal means, except for the castle–natural pair) (Figs. 4 and 5; SI Appendix Table S8). For δ^15^N values, similarities between these sites were weaker. According to ANOVA (or Welch *F* test), cats from rural sites differed too from those from other sites, except natural sites (SI Appendix Table S9). This result was the effect of much lower δ^15^N values in specimens from rural (and natural) locations.

Cats from seaports held a distinct position compared to other sites. These cats exhibited statistically significant different δ^15^N values to cats from other types of sites; except urban sites and castles. Their δ^15^N elevated values were attributed to higher contribution of marine food resources. For δ^13^C values, seaports statistically differed to strongholds, castles, and rural sites.

For the North Sea region, isotopic values significantly differed between most site types, in terms of both δ^13^C and δ^15^N (Figs. 4 and 5; SI Appendix Tables S10 and S11). In particular, there was a significant difference between urban sites and seaports (*p* < 0.001), which was not detected in the Baltic region (though, in the Baltic region, the null hypothesis of equal means was fulfilled for urban sites and seaports with weak probabilities, *p* < 0.15). The lowest δ^13^C and δ^15^N values were found at castle and rural sites, whereas the highest values related to seaports and a monastery located on the coast. The urban sites exhibited a wide range of values. The δ^15^N signal of single samples overlapped with the highest δ^15^N values detected at seaports, whereas the lowest signal fell in the range of castle and rural sites.

### Cat isotope signal vs. geomorphological setting

To detect geomorphological factors that might shape the isotope signal of cats, we grouped the sites into five categories: inland, river valley, lakeshore, sea coast, and lagoon/fjord (Fig. 4; Dataset S1; definitions presented in “Classification of the sites” section).

For cats from the Baltic region, we found no statistically significant difference between sites next to freshwater and brackish water bodies (lakes, lagoons, fjords, and larger rivers). This lack of differences was visible in terms of both δ^13^C and δ^15^N values (high *p*-values of the analysis of variance, usually *p* > 0.2, SI Appendix Tables S12 and S13). Inland and sea coast locations differed from each other and from all other geomorphological settings in terms of δ^13^C values. In contrast, δ^15^N values differed for certain pairings (e.g., inland vs. sea coast, inland vs. lagoon/fjord, sea coast vs. river valley, and sea coast vs. lagoon/fjord), with a weak similarity for inland vs. lakeshore pairs (p = 0.0535, SI Appendix Table S13). River valleys and inland settings had the broadest range of δ^15^N values (Fig. 5).

For the North Sea region, we detected statistically significant differences between most of site geomorphology categories in terms of both δ^13^C and δ^15^N values (analysis of variance *p* < 0.005, but was much lower in most pairs, SI Appendix Tables S14 and S15). The only statistically similar pair is sea coast vs. river valley, for δ^15^N values. However, *p* value for this pair is close to the 0.05 confidence level. Similar to the Baltic Sea region, cats from river valleys had the widest range of δ^15^N, as well as a wide range of δ^13^C values (Fig. 5d).

## Discussion

### Potential sources of variability in the stable isotope values of cats

In general, the carbon and nitrogen isotopic signal (δ^13^C and δ^15^N) of a carnivore is controlled by many factors. These include biological factors (choice of prey, spectrum of prey, and isotopic variability of prey) and indirect environmental factors, which are responsible for the isotopic signal in prey (connected to vegetation type, aquatic/terrestrial ecosystem, soil development stage, climate, altitude)^26,27,49–53^. For domestic carnivores, direct or indirect anthropogenic impacts might also play an important role in shaping isotopic signals. These include, but are not limited to, various human activities: (i) feeding on consumption/production waste or on selected food; (ii) creation of new landscapes, opening up habitats for new prey species; (iii) farming selected plants, which serve as a food base for herbivores (for pest rodents hunted by carnivores, or for domestic stock delivering meat and/or dairy that is further fed to carnivores); and (iv) supplying food that would otherwise be of limited availability to a terrestrial carnivore (such as food acquired from fishing or distant trade). The feeding behavior of cats is also driven by local conditions, including currently available food resources, their relationship with people (indoor, free-roaming, feral), and individual behavioral characteristics^1,4^. Recent studies of modern cat diet indicate high behavioral and feeding differentiation in domestic cats^1,17,18^.

### Terrestrial vs. aquatic resources

The most important factor shaping the isotope signal of the studied cats in the current study was the proportion of terrestrial and aquatic food resources in the diet. However, the regional isotope baseline (particularly the marine baseline), determined the degree to which differences could be detected through isotope values. In our dataset, the terrestrial and marine isotope signals were significantly different for cats from the North Sea region compared to those from the Baltic Sea region. Furthermore, the local background of human activity (especially cultivation of C_4_ plants and location of the fishing grounds and the type of fish exploited) could enhance or obscure the isotope indicators of consumed resources.

**Figure 6:**
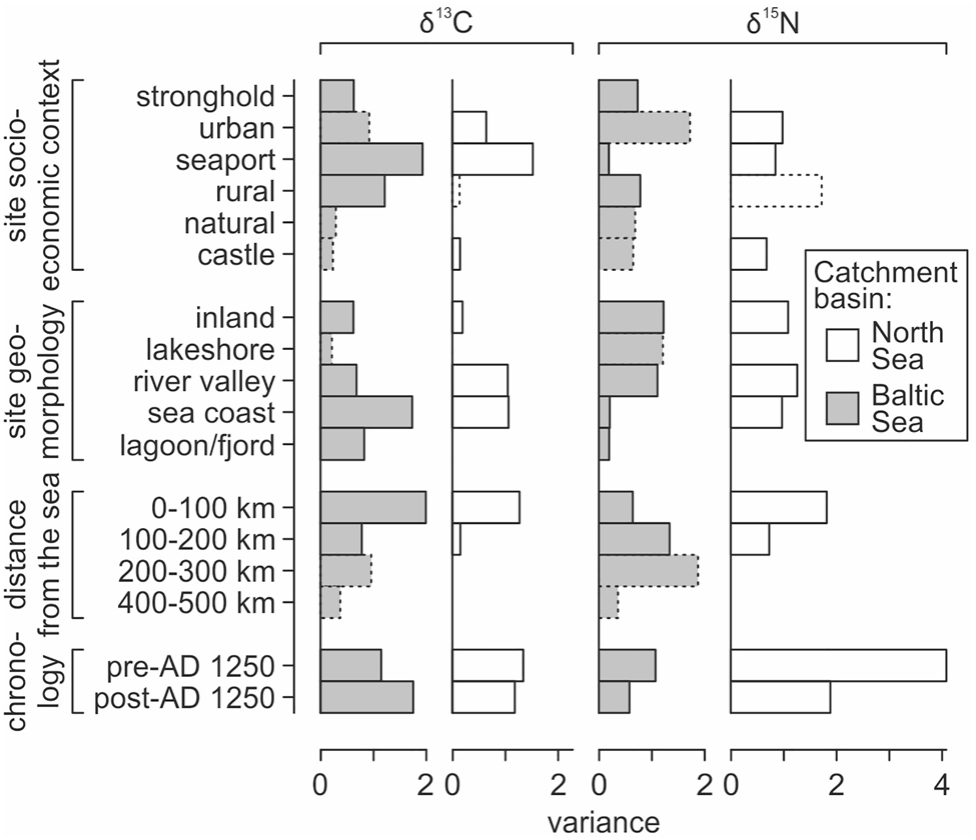
Variance (square of standard deviation) of δ^13^C and δ^15^N values in medieval and post-medieval cats grouped by site socio-economic context, site geomorphology, distance from the sea, and chronology. Dashed contour line used for groups with low number of specimens (n < 10). Groups with n < 3 (only North Sea region: monastery and 200–300 km distance from the sea) are not included. For detailed values see SI Appendix Tables S1, S4–S7.

Cats from variable locations situated near water bodies (both marine and freshwater: sites on the sea coast, lagoons/fjords, river valleys, and lakeshores) had similar isotopic patterns with high δ^15^N values, whereas inland (i.e., far from the water bodies) cats had lower δ^15^N values (Figs. 5d and 6). Such elevated δ^15^N values are typical for aquatic ecosystems^53^. Thus, water-derived food resources contributed significantly to the diets of cats close to water bodies. Considering the socio-economic context of sites, higher δ^15^N values were expected in cats from seaports (Fig. 5b). Elevated δ^15^N values are also typical for cats from castles and strongholds in the Baltic region (Figs. 5b and 6). This is due to the cross-effect of cultural function and geomorphological setting of these sites, which were usually defensive locations situated on the edge of lakes or rivers (23 out of 25 of these sites in our database were attributed to river valley, lakeshore, or lagoon/fjord).

Cats from non-coastal sites near to water (i.e., lakeshores, river valleys, and lagoons/fjords) were statistically dissimilar from those on the sea coast. This result was due to slightly lower mean δ^15^N values and clearly lower minimum δ^15^N values (Fig. 5d). This finding supported the expected difference between freshwater and marine fish^54,55^. In parallel, much lower δ^13^C values were obtained for cats from near-water non-coastal sites compared to cats from seashores (Fig. 5c). Despite being located quite far from the seashore, Bremen did not follow this relationship, as it had marine-like δ^13^C and δ^15^N values. However, availability of the marine resources in Bremen might have been due to the towns' trade hub function (see further discussion in “Cat isotope signal and the level of urbanization” and “Local conditions” sections).

The nitrogen and carbon isotopic signals in cats suggest an opportunistic shift toward local aquatic resources when available and a high reliance on such resources. This phenomenon might be related to scavenging fish remains or cats hunting terrestrial piscivores. Such prey might include sea birds, common in port towns and characterized by a marine-like δ^15^N signal^56^, and omnivorous rodents, which forage on fishery wastes and exhibit elevated δ^15^N values in port towns^57,58^.

### Cat isotope signal and the level of urbanization

Cats from urban sites and seaports had the most varied δ^13^C values (Figs. 5a and 6). Cats from seaports had the highest mean δ^15^N values and were characterized by the lowest variability in δ^15^N (Figs. 5b and 6). These differences in δ^13^C and δ^15^N values variability indicated different primary food sources. The nitrogen signal is mainly related to the input of terrestrial (low δ^15^N values) vs. aquatic (high δ^15^N values) resources. The carbon isotopic signal depends on marine (high δ^13^C values, but there are other differences between littoral and pelagic ecosystems) vs. non-marine (terrestrial and freshwater) resources. The presence of C_4_ plants might also elevate the latter. The observed low δ^15^N and high δ^13^C variation in seaport cats indicated that aquatic resources were an important component of their diet, with mixed marine and non-marine resources, or mixed marine-littoral and marine-pelagic resources, or both.

The diet of cats from coastal locations was mainly driven by marine-derived food, whereas that of urban cats included a greater variety of food resources. Urban cats had high mean δ^15^N, but also, highly varied δ^15^N values (Fig. 5b). However, high-δ^15^N values of freshwater and/or marine food was still available to urban cats, regardless of whether it was naturally present in the local ecosystem. This finding demonstrates the importance of anthropogenic resources for these cats, as the availability of water-derived food was certainly due to trade. Medieval logistics allowed transport of fresh sea-fish up to 150 km inland^59^. This heterogeneity in the diet of cats in urban areas has also been identified in studies of modern cats^11,32,58,60,61^: cats consume more anthropogenic food in urban habitats, while predation rate and prey diversity are highest in rural areas.

Cats connected to natural locations exhibited relatively low variability for both carbon and nitrogen (Fig. 5a). They were characterized by low δ^15^N and δ^13^C values, which is typical for forest animals^10,34^. This observation indicates a narrow isotopic niche and trophic specialization, and shows that cats probably mainly inhabited forests and exploited wild prey. Studies of recent cats also support this explanation, showing that feral domestic cats tend to have a narrower trophic niche than house cats, with their diet similar to that of wildcats^18^.

The carbon and nitrogen isotopic ranges of rural cats overlapped with those from natural sites (Fig. 5a,b). However, rural cats exhibited a broader range of values, especially for δ^13^C. Like cats from natural sites, rural cats had lower δ^13^C values, and the lowest mean δ^15^N values, compared to other locations. These results indicate the use of just terrestrial prey resources, and predation over wild prey species. Increased predation rates in rural areas commonly occurs in modern cats^11,34,60,61^. While urban cats are more likely to be locked inside or living close to households, rural cats have lower population densities but have more extensive home ranges and live closer to natural habitats^11,17,18,62,63^.

### Local conditions

Detailed analysis of our dataset allowed us to detect some abnormalities in the isotopic pattern. For instance, several sites from the Baltic region (Kołobrzeg, Puck, Napole, and Visby) stood out from the whole dataset due to their high δ^13^C signal (Fig. 4; Datasets S1 and S2). Several potential factors could be responsible for such a high δ^13^C signal: low forestation^26^, high elevation^64,65^, or significant contribution of marine food resources^54,55^. Another factor that elevates δ^13^C values might be attributed to the important contribution of C_4_ plants (e.g., millet) at the basis of the trophic web. These C_4_ plants were possibly stored cereals, which served as a source of food for pest rodents, which, in turn, were preyed upon by cats. The presence of millet was confirmed in archaeobotanical material from Puck^66^. However, in the case of coastal sites, such as Puck, Kołobrzeg and Visby, the marine resources could also play an important role. Cats had significantly different δ^15^N values in these seaports versus Napole. These seaports had the highest, whereas Napole had the lowest values out of the entire dataset. This difference followed the seaport vs. rural site function, and likely reflected differences in the proportion of marine food available at each site.

Another interesting example concerns fortified sites, namely strongholds and castles from the Baltic region. Cats from these sites exhibited the lowest isotopic variability for δ^13^C values and moderate variability for δ^15^N values. The absolute δ^13^C values were similar to natural and rural sites, whereas δ^15^N values were usually higher (Fig. 4). This result indicates that freshwater fish formed an important component of the diet, which is logical considering the location of these sites (23 out of 25 sites were attributed to the river valley, lakeshore, or lagoon/fjord geomorphological setting). Cats inside these closed settlements might have preferred living close to humans, and therefore had an easy access to table refuse.

The castle of Logne was the only castle situated in the North Sea region in our database. Logne cats had a clearly lower δ^15^N signal compared to cats from castles in the Baltic Sea region (Fig. 4). This result was unexpected when considering the generally higher δ^15^N values baseline in the North Sea region. Cats from this location (which is rather far from the coast), might have had limited access to fish and other water resources. The region has been densely forested until recent times, which suggests that the Logne cats could have had easy access to forest resources (via hunting or access to the table refuse of game animal meat), reflected in their low δ^15^N and δ^13^C values.

The opposite situation is visible in cats from Bremen, recording marine-like isotopic signals despite its location in the freshwater zone of the Weser^67^. Marine fish, however, were commonly available in Bremen as evidenced by zooarchaeological studies and historical records. Some anadromous species (e.g., sturgeon, salmon, sea trout, smelt, twaite shad, and flounder) used to enter the Weser during their spawning season and might have been fished locally^68^. The Bremen fishermen were organized in a guild from at least 1489, which held the fishing rights down to the river mouth^69^. Additionally, Bremen was an important trade hub since at least the eleventh century, particularly involved in the stockfish and herring trade^70^.

### A model of cat isotopic ecology

**Figure 7:**
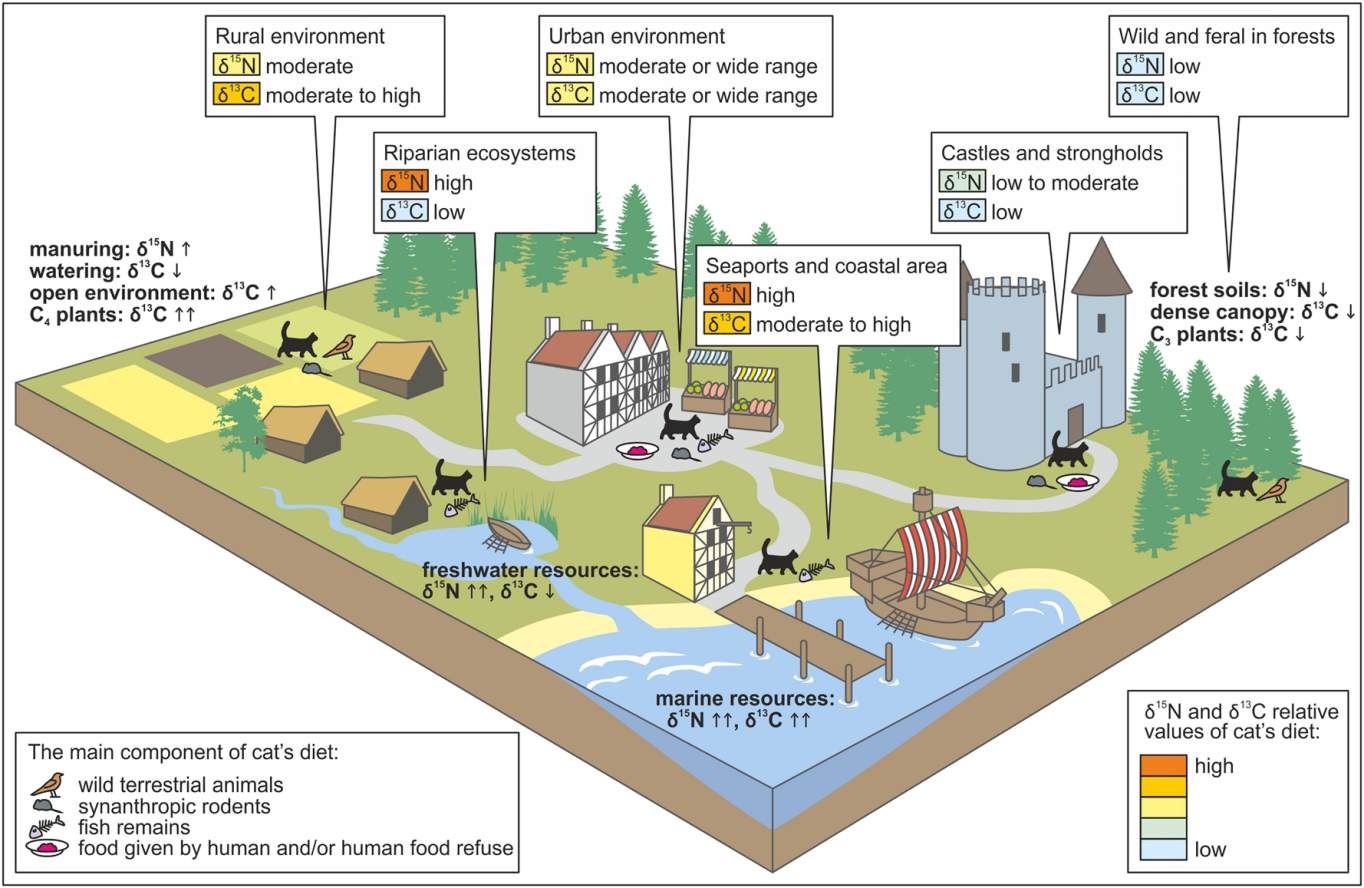
Concept of the variability of carbon and nitrogen isotope signal in cats in typical medieval and post-medieval anthropogenic landscapes.

In general, domestic cats may follow three main foraging strategies: (i) hunting, (ii) scavenging, mainly on human waste, and iii) direct feeding by humans. Considering these strategies, we may define the following factors responsible for an isotopic signal of cat diet, synthesized in Fig. 7:

- Presence of cereal crops, cultivated in open fields, characterized by elevated δ^13^C values^26,27^, and available to synanthropic rodents (such as mice and rats, hunted by cats) either directly from fields, storages in barns or granaries, or from processed products;
- Cultivation or import of C_4_ cereal crops with high δ^13^C signals (such as millet or maize)^50,71^;
- Agricultural treatments, which may influence isotopic values of cultivated plants, such as manuring (elevating δ^15^N values in crops, especially in grains) and watering (lowering δ^13^C values in crops)^49,51,52,72^;
- The proximity of forests, where cats may hunt for wild animals characterized by low δ^13^C and low δ^15^N values in natural habitats, due to canopy effect and forest soils^26,73^;
- Access to meat/dairy from domestic animals that forage on agriculturally treated or untreated food (manured/not manured, watered/not watered, with/without C_4_ plants), and access to the meat of wild animals (provided by huntsmen, poachers, and, during medieval times, especially by knights and noblemen who were allowed to hunt in forests)^74^;
- Access to fish (and other marine resources), usually characterized by an elevated δ^15^N signal and variable δ^13^C values, depending on fish ecology and physiology (i.e., differences between freshwater and marine fish^54,55^, geography (e.g., the difference between the Baltic Sea and North Sea^47,48^, or between pelagic and littoral waters^53^).

In a human-dominated landscape, the isotopic signal of cat diet can be impacted by all of the listed factors. However, certain basic patterns related to variable anthropogenic and geographical settings can be predicted. In rural areas, crop pests are easy prey; however, the proximity of natural environments encourages the opportunity for cats to wander and feed on wild prey. Modern rural cats have a more diverse diet and a higher predation rate compared to those inhabiting urban sites^11,60,61^. Towns favor larger populations of synanthropic rodents, due to food stores and refuse. In addition, urban areas as trading spots provide various imported food resources, including marine fish, even in inland locations. However, even the largest inland towns cannot compete with seaports in terms of the availability of marine resources.

## Conclusions

The ecology of the domestic cat is complex because it is an opportunistic exploiter that can maneuver among multiple available ecological niches^1^. Modern cats sharing the same environment, especially town-dwellers, exploit highly variable habitats and, hence, have highly varied trophic ecology^1,12,16,17,31,72^. Some cats may live as "backyard cats," (i.e., welcome but free-roaming mouse hunters), which are occasionally fattened by their human neighbors. Others might be "feral outcasts," which are the barely tolerated semi-wild dwellers of cellars, roofs, and hovels, living on their own and avoiding human contact. Still others might live as "indoor pets." Some cats even, exploit all these lifestyles.

The individualistic behavior and the taphonomic limitations mean that the isotopic signature of a single fossil cat specimen, or even of several specimens, might not represent the entire population. These limitations were revealed in our study through the high variability of the isotopic signal within the cats from some socio-economic types of sites (particularly those from seaports, urban and rural settlements) and geomorphological settings (especially river valleys and sea coasts). However, our data also revealed relatively low isotopic variability in cats from specific locations (mainly natural sites, outside of cultural context). This phenomenon most likely relates to feral cats, which can permanently or temporarily adopt a wildcat-like lifestyle. Similarly to modern cat populations, the medieval cats tended to have more heterogenous diet in urban areas, as they consumed more anthropogenic food. At the same time, the predation rate was higher in rural and natural areas.

The observed isotopic diversity means that stable isotope analysis could be a useful tool in elucidating the diet and lifestyle of individual cats. However, a local/regional isotopic baseline must always be carefully considered, as no global range may be arbitrarily attributed to the particular ecology of a cat. This study shows a promising perspective concerning the trophic ecology of fossil and recent cats, offered by an analysis of diet-related stable isotopes preserved in bones. Further investigations using sulfur stable isotope analysis and compound-specific carbon and nitrogen isotope analysis may deliver more in-depth insight into the domestic cat ecology.

## Materials and methods

### Zooarchaeological material

We collected the bones of small felids from archaeological and paleontological sites located in Poland, Germany, and Belgium (Fig. 1; Datasets S1 and S2). Our dataset included various geographical locations, covering human settlements of various socio-economic contexts, and natural sites. The available remains were either single bone specimens or almost complete skeletons. In total, 190 bones originating from 60 archaeological sites at 40 locations were sampled. The characteristics of the sampled specimens and sites included in this study are listed in (Datasets S1 and S2). We also included literature data of 18 felid bones from Sweden, Norway, UK, Belgium and Germany, from previously published studies^38–45^ (Dataset S3).

Bones most probably belonging to the same individual (based on: the same stratigraphic unit; different skeletal elements; similar size, individual age and preservation state), were sampled and analyzed separately, but their isotopic values were averaged (Dataset S4).

#### Taxonomic identification—domestic cat vs. European wildcat

Preliminary identification to the genus level (*Felis* sp. Linnaeus, 1758) was carried out in the Royal Belgian Institute of Natural Sciences and the Institute of Archaeology, Nicolaus Copernicus University in Toruń, using reference collections of domestic cat and European wildcat skeletons. The bones were measured according to the methodology by A. von den Driesch^75^, using a digital caliper (0.1 mm reading error) (Dataset S2). To determine the species (domestic cat, *F. catus* Linnaeus, 1758 vs. wildcat, *F. silvestris* Schreber, 1777, considered here to be European wildcat, *F. silvestris silvestris*) we adopted the discrimination method based on relative size using the log-ratio technique by T. P. O'Connor^76^ (Datasets S5, S6 and S7). The reference standards for domestic cat and European wildcat size ranges were based on Z. Kratochvíl's database^77,78^.

Due to limitations in distinguishing domestic cat bones from the European wildcat based on osteometric and morphological features, we also used mtDNA analysis, especially for specimens where the size ranges of domestic cats and European wildcats overlapped (SI Appendix Fig. S3; Dataset S2).

On the basis of morphometry and/or mtDNA analysis, we distinguished 139 domestic cats and 12 European wildcats. The specimens for which we could not obtain genetic or morphometric taxonomical information (n = 31) likely represent domestic cats due to their relatively small size, and have been regarded as such in further analyses. Specimens extracted from previous publications (n = 18) were also considered domestic cats.

#### Cat age criteria

We selected bones of individuals that were older than the nursing period, to avoid the milk diet obscuring trophic ecology of individuals. Cat dependence on the mother's milk is short and weaning is considered finished by about 7 weeks after birth^1^. Thus, for our study we selected adult individuals (with bone fusion completed) and subadult individuals, i.e. older than weaning age, based on combined bone fusion or tooth eruption^79^. For fragmented bones, when it was not possible to apply the criteria above, we used overall bone size and stage of bone ossification as indicators of post-weaning age (Dataset S2).

### Classification of the sites

#### Chronology

The chronology of the specimens was presented as ranges in years AD. These ranges were based on the radiocarbon determination of the specimen if available, or/and on stratigraphic and archaeological dating (Datasets S1 and S2), using the published data (and in some cases, unpublished archaeological reports) (Dataset S2).

We used all radiocarbon dates available in the published literature for the previously studied specimens^8,10,80^, and new data obtained during the course of current projects by the authors (Dataset S2). In all cases, the dated fraction used was bone collagen. Most dates were obtained in the Poznań Radiocarbon Laboratory (Poznań, Poland), while some dates were obtained in the Royal Institute for Cultural Heritage (RICH, Brussels, Belgium). The presented chronology range was the end points of the 95.4% probability range, achieved through calibration in OxCal, v. 4.4^81,82^. The ranges were provided with a ± 1-year accuracy.

The resolution varied among sites, from intervals of a part of a century to several centuries-long. We attributed chronological ranges to the stratigraphic data provided as centuries or parts of centuries in the following way:

- a century: AD 1X00–1Y00;
- early century: AD 1X00–1X25;
- late century: AD 1X75–1Y00;
- mid-century: AD 1X50;
- 1st half of a century: AD 1X00–1X50;
- 2nd half of a century: AD 1X50–1Y00.

On rare occasions the chronological data were more precise (i.e., narrowed to a period before or after a particular year). In such cases, we used these years as the range end. In cases where chronology was provided in a descriptive way, we adopted the following scheme: late medieval = AD 1250–1500; post-medieval = AD 1500–1800.

#### Geomorphology

We attributed a geomorphological context to the studied sites based on their geographic location (Dataset S1). We used the following types of geomorphological setting:

- sea coast (less than 5 km from the coast of a sea or a bay; taking into account home size ranges and mobility to acquire food^1,83,84^);
- lagoon/fjord (over 5 km from an open sea coast, but less than 5 km from a brackish basin, such as a lagoon or fjord, which was weakly connected with the open sea);
- lakeshore (less than 5 km from a larger lake, or at an island);
- river valley (within a valley or at the edge of a valley of a large river);
- inland (none of the above, i.e., over 5 km from the nearest sea coast, brackish basin, lake or large river valley).

#### Site socio-economic context

We attributed a socio-economic context to each site based on data from the published literature (Dataset S1), also taking into account the chronology of the studied specimens for sites where the function changed during medieval/post-medieval times. We attributed the following functions:

- natural (no historical nor archaeological indications for any anthropogenic use of the site during the considered period; mostly cave sites);
- rural (small settlement, possibly farmland, often loosely constructed with wooden buildings);
- urban (towns, administrative centers, densely built-up, densely populated);
- seaport (important marine ports; all of them were also urban sites); in this category, we also include Bremen, which was involved in marine fishing and trade in the past^68–70^;
- castle (late medieval fortified masonry seat of a noble family, a bishop, or knights of the Teutonic Order);
- stronghold (mostly early medieval fortified settlement or refuge with wooden and earthen embankments; only in the eastern part of the studied area);
- monastery (late medieval ecclesiastic settlement; only one example, Koksijde Monastery in Belgium).

#### Latitude, longitude, and distance from the sea

For cave sites, we provided the geographic coordinates of cave entrances. For open-air sites, we provided the coordinates of the center of a village/small town, center of a castle, or center of a street. We used Google Maps to obtain coordinates (decimal N and E values, ± 0.00001°). To provide the distance from the sea, we used the "measure distance" option in Google Maps. We measured this as the shortest distance from the coordinate-collecting point to the nearest sea coast line (± 1 km).

### Stable isotope analysis

The bulk stable isotope analysis of carbon and nitrogen was performed on collagen extracted from bones. Dentine was not sampled to avoid bias from inter-tissue fractionation.

For samples with lab. no. CAT, collagen purification method followed the protocol after Krajcarz et al. and Bocherens et al.^10,85^. Isotopic measurements (δ^13^C and δ^15^N) and elemental measurements (elemental C:N ratio, calculated into atomic C:N ratio) were performed in duplicate at the Stable Isotopes Laboratory at the Institute of Geological Sciences, Polish Academy of Sciences (Warszawa, Poland) using a Flash EA 1112HT elemental analyzer (Thermo Scientific) connected to a Delta V Advantage mass spectrometer (Thermo Scientific). Mean SEs were < 0.33‰ for δ^13^C and < 0.43‰ for δ^15^N values. We normalized all measurements to δ^13^C values of USGS40 and USGS41 standards and all δ^15^N values to the IAEA 600 standard. Details are provided in SI Appendix Supplementary Text and Table S16.

For all of the remaining samples, collagen extraction was carried out at the Royal Institute for Cultural Heritage (RICH) in Brussels. Samples were prepared according to the method of Longin^86^, which was modified with an additional NaOH-wash between demineralization and hydrolyzation^87,88^. Collagen subsamples were sent to the Division of Soil and Water Management of the KU Leuven for stable isotope (δ^13^C and δ^15^N) and elemental (%C, %N) analyses. The atomic C:N ratio was then calculated. Analyses were carried out on a Thermo Flash HT/EA elemental analyzer, linked to a Thermo DeltaV Advantage Isotope Ratio Mass Spectrometer via a ConfloIV interface (Thermo Scientific). Data calibrations were done using IAEA-600 (caffeine) and two in-house standards (Leucine and muscle tissue of Pacific Tuna), which were previously calibrated versus certified standards. Analytical precision was typically better than 0.15‰ for both δ^13^C and δ^15^N values, this was based on the average of four replicates of the different standards in each batch of samples.. Details are provided in SI Appendix Supplementary Text and Table S16.

Data were reported in delta (δ) notation: δ^13^C or δ^15^N = ([R_sample_/R_international reference_] – 1) × 1000 in ‰ (parts per thousand), where R is ^13^C/^12^C or ^15^N/^14^N. The international references were Vienna Pee Dee Belemnite (VPDB) for carbon and atmospheric nitrogen (AIR) for nitrogen.

### Statistics

We used one-way Analysis of Variance (ANOVA; or alternatively Kruskal–Wallis or Welch *F* tests in the case of non-parametric distributions or unequal variances) to check for statistical differences between isotopic variances of selected groups of samples (such as samples representing: chronological intervals, site cultural functions or site geomorphologies). The homogeneity of variance and normality were tested with Levene's tests (from means and medians) and a set of normality tests (Shapiro–Wilk, Anderson–Darling, Lilliefors and Jarque–Bera tests)^89^. Because in many cases the groups exhibited non-normal distribution and unequal variances, post-hoc pairwise comparisons were difficult to perform. Therefore, analysis of variance was run separately for each pair. In cases where post-hoc tests were possible for the entire set of pairs, we applied Tukey's test (after a significant ANOVA) with the Bonferroni correction of *p* values for multiple comparisons. All tests were performed with PAST software, v. 4.05^90^.

### Methods of ancient DNA analysis

Genetic analyses of 75 samples were performed at the Centre of New Technologies at the University of Warsaw, Poland. All samples were analyzed in facilities dedicated to working with ancient DNA following strict rules to avoid contamination. Samples were prepared and processed as described by Baca et al.^8^. DNA was extracted using silica-coated magnetic beads according to Rohland et al.^91^. Double-strand, double-indexed library preparation, target enrichment of mitochondrial DNA (mtDNA), sequencing on the Illumina platform, and processing of the sequencing reads were performed as in Baca et al.^8^, with the modification that only positions with a minimum of 3× coverage were called. We obtained mtDNA sequences for 57 specimens, which were used in phylogenetic analysis, along with 123 already published sequences^5,8,10^. The Maximum Likelihood Tree was generated using IQtree^92^. The implemented ModelFinder determined that GTR is the best-fit model and the branch support was assessed using ultrafast bootstrap method (SI Appendix Fig. S3). All mtDNA sequences obtained in this study were deposed in GenBank under accession numbers: OL654306–OL654362.

## Supplementary Information

Supplementary Information 1.

Supplementary Information 2.

## Data Availability

All data are available in the main text or the supplementary materials. DNA sequences obtained in this study were deposited in GenBank under accession numbers: OL654306–OL654362; direct link: https://www.ncbi.nlm.nih.gov/popset/?term=2259973155.

## References

[1] Turner D, Bateson P (2000). The Domestic Cat: The Biology of Its Behaviour.

[2] Bradshaw JWS, Goodwin D, Legrand-Defrétin V, Nott HMR (1996). Food selection by the domestic cat, an obligate carnivore. Comp. Biochem. Physiol. A Physiol..

[3] Trouwborst A, McCormack PC, Martínez Camacho E (2020). Domestic cats and their impacts on biodiversity: A blind spot in the application of nature conservation law. People Nat..

[4] Crowley SL, Cecchetti M, McDonald RA (2020). Our wild companions: Domestic cats in the anthropocene. Trends Ecol. Evol..

[5] Driscoll CA (2007). The Near Eastern origin of cat domestication. Science.

[6] Van Neer W, Linseele V, Friedman R, De Cupere B (2014). More evidence for cat taming at the Predynastic elite cemetery of Hierakonpolis (Upper Egypt). J. Archaeol. Sci..

[7] Ottoni C (2017). The palaeogenetics of cat dispersal in the ancient world. Nat. Ecol. Evol..

[8] Baca M (2018). Human-mediated dispersal of cats in the Neolithic Central Europe. Heredity.

[9] Vigne J (2019). The beginning of cat domestication in East and West Asia. Doc. Archaeobiol..

[10] Krajcarz M (2020). Ancestors of domestic cats in Neolithic Central Europe: Isotopic evidence of a synanthropic diet. Proc. Natl. Acad. Sci. USA.

[11] Piontek AM (2021). Analysis of cat diet across an urbanisation gradient. Urban Ecosyst..

[12] Medina FM (2011). A global review of the impacts of invasive cats on island endangered vertebrates. Glob. Chang. Biol..

[13] Moseby KE, Peacock DE, Read JL (2015). Catastrophic cat predation: A call for predator profiling in wildlife protection programs. Biol. Conserv..

[14] Loss SR, Will T, Marra PP (2013). The impact of free-ranging domestic cats on wildlife of the United States. Nat. Commun..

[15] Beaumont M (2001). Genetic diversity and introgression in the Scottish wildcat. Mol. Ecol..

[16] Beugin MP (2020). Hybridization between *Felis silvestris silvestris* and *Felis silvestris catus* in two contrasted environments in France. Ecol. Evol..

[17] Biró Z, Lanszki J, Szemethy L, Heltai M, Randi E (2005). Feeding habits of feral domestic cats (*Felis catus*), wild cats (*Felis silvestris*) and their hybrids: Trophic niche overlap among cat groups in Hungary. J. Zool..

[18] Széles GL, Purger JJ, Molnár T, Lanszki J (2018). Comparative analysis of the diet of feral and house cats and wildcat in Europe. Mammal. Res..

[19] Ottoni C, Van Neer W (2020). The dispersal of the domestic cat paleogenetic and zooarcheological evidence. Near East. Archaeol..

[20] Bitz-Thorsen J, Gotfredsen AB (2018). Domestic cats (*Felis catus*) in Denmark have increased significantly in size since the Viking Age. Danish J. Archaeol..

[21] Faure E, Kitchener AC (2009). An archaeological and historical review of the relationships between felids and people. Anthrozoos.

[22] von den Driesch A, Schmidt V, Horzinek MC (1992). Kulturgeschichte der Hauskatze. Krankheiten der Katze, Bd. 1.

[23] Głażewska I, Kijewski T (2017). A new view on the European feline population from mtDNA analysis in Polish domestic cats. Forensic Sci. Int. Genet..

[24] Cucchi T (2020). Tracking the Near Eastern origins and European dispersal of the western house mouse. Sci. Rep..

[25] Van Klinken GJ, Richards MP, Hedges BEM, Ambrose S, Katzenberg M (2002). An overview of causes for stable isotopic variations in past European human populations: environmental, ecophysiological, and cultural effects. Biogeochemical Approaches to Paleodietary Analysis.

[26] Drucker DG, Bridault A, Hobson KA, Szuma E, Bocherens H (2008). Can carbon-13 in large herbivores reflect the canopy effect in temperate and boreal ecosystems? Evidence from modern and ancient ungulates. Palaeogeogr. Palaeoclimatol. Palaeoecol..

[27] Koch PL, Michener R, Lajtha K (2007). Isotopic study of the biology of modern and fossil vertebrates. Stable Isotopes in Ecology and Environmental Science.

[28] Hofman-Kamińska E (2018). Foraging habitats and niche partitioning of European large herbivores during the holocene—Insights from 3D dental microwear texture analysis. Palaeogeogr. Palaeoclimatol. Palaeoecol..

[29] Bocherens H, Hofman-Kamińska E, Drucker DG, Schmölcke U, Kowalczyk R (2015). European bison as a refugee species? Evidence from isotopic data on Early Holocene bison and other large herbivores in northern Europe. PLoS ONE.

[30] Hu Y (2014). Earliest evidence for commensal processes of cat domestication. Proc. Natl. Acad. Sci. USA..

[31] Haruda AF (2020). The earliest domestic cat on the Silk Road. Sci. Rep..

[32] Meckstroth AM, Miles AK, Chandra S (2007). Diets of introduced predators using stable isotopes and stomach contents. J. Wildl. Manag..

[33] McDonald BW (2020). High variability within pet foods prevents the identification of native species in pet cats’ diets using isotopic evaluation. PeerJ.

[34] Maeda T, Nakashita R, Shionosaki K, Yamada F, Watari Y (2019). Predation on endangered species by human-subsidized domestic cats on Tokunoshima Island. Sci. Rep..

[35] Stewart GR, Aidar MPM, Joly CA, Schmidt S (2002). Impact of point source pollution on nitrogen isotope signatures (δ^15^N) of vegetation in SE Brazil. Oecologia.

[36] Graven H, Keeling RF, Rogelj J (2020). Changes to carbon isotopes in atmospheric CO_2_ over the industrial era and into the future. Glob. Biogeochem. Cycles.

[37] DeNiro MJ (1985). Postmortem preservation and alteration of in vivo bone collagen isotope ratios in relation to palaeodietary reconstruction. Nature.

[38] Linderholm A, Kjellström A (2011). Stable isotope analysis of a medieval skeletal sample indicative of systemic disease from Sigtuna Sweden. J. Archaeol. Sci..

[39] Webb EC (2018). Compound-specific amino acid isotopic proxies for distinguishing between terrestrial and aquatic resource consumption. Archaeol. Anthropol. Sci..

[40] Müldner G, Richards MP (2007). Stable isotope evidence for 1500 years of human diet at the city of York, UK. Am. J. Phys. Anthropol..

[41] Müldner G, Richards MP (2005). Fast or feast: Reconstructing diet in later medieval England by stable isotope analysis. J. Archaeol. Sci..

[42] van der Sluis LG, Hollund HI, Kars H, Sandvik PU, Denham SD (2016). A palaeodietary investigation of a multi-period churchyard in Stavanger, Norway, using stable isotope analysis (C, N, H, S) on bone collagen. J. Archaeol. Sci. Rep..

[43] Polet C, Katzenberg MA (2003). Reconstruction of the diet in a mediaeval monastic community from the coast of Belgium. J. Archaeol. Sci..

[44] Kosiba SB, Tykot RH, Carlsson D (2007). Stable isotopes as indicators of change in the food procurement and food preference of Viking Age and Early Christian populations on Gotland (Sweden). J. Anthropol. Archaeol..

[45] Olsen KC (2018). Isotopic anthropology of rural German medieval diet: Intra- and inter-population variability. Archaeol. Anthropol. Sci..

[46] Benevolo L (1993). The European City.

[47] Barrett J (2008). Detecting the medieval cod trade: A new method and first results. J. Archaeol. Sci..

[48] Barrett JH (2011). Interpreting the expansion of sea fishing in medieval Europe using stable isotope analysis of archaeological cod bones. J. Archaeol. Sci..

[49] Bogaard A, Heaton THE, Poulton P, Merbach I (2007). The impact of manuring on nitrogen isotope ratios in cereals: Archaeological implications for reconstruction of diet and crop management practices. J. Archaeol. Sci..

[50] Heaton THE (1999). Spatial, species, and temporal variations in the ^13^C/^12^C ratios of C3 plants: Implications for palaeodiet studies. J. Archaeol. Sci..

[51] Bogaard A (2013). Crop manuring and intensive land management by Europe’s first farmers. Proc. Natl. Acad. Sci. USA..

[52] Styring AK (2015). Refining human palaeodietary reconstruction using amino acid δ^15^N values of plants, animals and humans. J. Archaeol. Sci..

[53] Guiry E (2019). Complexities of stable carbon and nitrogen isotope biogeochemistry in ancient freshwater ecosystems: Implications for the study of past subsistence and environmental change. Front. Ecol. Evol..

[54] Fuller BT, Müldner G, Van Neer W, Ervynck A, Richards MP (2012). Carbon and nitrogen stable isotope ratio analysis of freshwater, brackish and marine fish from Belgian archaeological sites (1st and 2nd millennium AD). J. Anal. At. Spectrom..

[55] Robson HK (2016). Carbon and nitrogen stable isotope values in freshwater, brackish and marine fish bone collagen from Mesolithic and Neolithic sites in central and northern Europe. Environ. Archaeol..

[56] Hobson KA, Piatt JF, Pitocchelli J (1994). Using stable isotopes to determine seabird trophic relationships. J. Anim. Ecol..

[57] Guiry E, Buckley M (2018). Urban rats have less variable, higher protein diets. Proc. R. Soc. B Biol. Sci..

[58] Bicknell AWJ (2020). Stable isotopes reveal the importance of seabirds and marine foods in the diet of St Kilda field mice. Sci. Rep..

[59] Hoffmann RC, Squatriti P (2000). Medieval fishing. Working with Water in Medieval Europe. Technology and Resource-Use.

[60] Gillies C, Clout M (2003). The prey of domestic cats (*Felis catus*) in two suburbs of Auckland City, New Zealand. J. Zool..

[61] Brickner-Braun I, Geffen E, Yom-Tov Y (2007). The domestic cat as a predator of Israeli wildlife. Isr. J. Ecol. Evol..

[62] Flockhart DTT, Norris DR, Coe JB (2016). Predicting free-roaming cat population densities in urban areas. Anim. Conserv..

[63] Castañeda I, Zarzoso-Lacoste D, Bonnaud E (2020). Feeding behaviour of red fox and domestic cat populations in suburban areas in the south of Paris. Urban Ecosyst..

[64] Zhu Y, Siegwolf RTW, Durka W, Körner C (2010). Phylogenetically balanced evidence for structural and carbon isotope responses in plants along elevational gradients. Oecologia.

[65] Männel TT, Auerswald K, Schnyder H (2007). Altitudinal gradients of grassland carbon and nitrogen isotope composition are recorded in the hair of grazers. Glob. Ecol. Biogeogr..

[66] Pińska K, Badura M, Starski M (2017). Warunki przyrodnicze i dieta roślinna mieszkańców Pucka w późnym średniowieczu. Puck - kultura materialna małego miasta w późnym średniowieczu.

[67] Lefebvre A (2022). Morphology of estuarine bedforms, Weser Estuary, Germany. Earth Surf. Process. Landforms.

[68] Bischop D, Von der Küchelmann HC, Melzer W (2018). Küche in den Graben – Bremens Stadtgraben und die Essgewohnheiten seiner Anwohner an der Wende zur Frühen Neuzeit.. Lebensmittel im Mittelalter und in der frühen Neuzeit. Erzeugung, Verarbeitung, Versorgung. Beiträge des 16. Kolloquiums des Arbeitskreises zur archäologischen Erforschung des mittelalterlichen Handwerks, Soester Beiträge zur Archäologie 15.

[69] Elmshäuser K, Pordzik VV (2019). Lachsgarnen, Tomen und Kumpanen – Die älteste Bremer Fischeramtsrolle. Bremisches Jahrb..

[70] Küchelmann HC, Kahlow S, Schachtmann J, Hähn C (2021). Viel Butter bei wenig Fisch. Zwei Fischknochenkomplexe des 12.–13. Jahrhunderts aus der Bremer Altstadt.. Grenzen überwinden. Archäologie zwischen Disziplin und Disziplinen. Festschrift für Uta Halle zum 65. Geburtstag, Internationale Archäologie Studia Honoraria 40.

[71] Schwarcz HP, Schoeninger MJ (1991). Stable isotope analyses in human nutritional ecology. Am. J. Phys. Anthropol..

[72] Wallace M (2013). Stable carbon isotope analysis as a direct means of inferring crop water status and water management practices. World Archaeol..

[73] van der Merwe NJ, Medina E (1991). The canopy effect, carbon isotope ratios and foodwebs in amazonia. J. Archaeol. Sci..

[74] Ervynck A, O’Day SJ, Van Neer W, Ervynck A (2004). Orant, pugnant, laborant. The diet of the three orders in the feudal society of medieval north-western Europe. Behaviour Behind Bones. The Zooarchaeology of Ritual, Religion, Status and Identity.

[75] von den Driesch A (1976). A guide to the measurement of animal bones from archaeological sites. Peabody Museum Bull..

[76] O’Connor TP (2007). Wild or domestic? Biometric variation in the cat *Felis silvestris* Schreber. Int. J. Osteoarchaeol..

[77] Kratochvíl Z (1973). Schadelkriterien der Wild- und Hauskatze (*Felis silvestris silvestris* Schreber 1777 und *Felis s.* f. catus L. 1758). Acta Sci. Nat. Brno.

[78] Kratochvíl Z (1976). Das Postkranialskelett der Wild- und Hauskatze (*Felis silvestris* und *F. lybica* f. catus). Acta Sci. Nat. Brno.

[79] Dyce KM, Sack WO, Wensing CJG (2010). Textbook of Veterinary Anatomy.

[80] Krajcarz M (2016). On the trail of the oldest domestic cat in Poland. An insight from morphometry, ancient DNA and radiocarbon dating. Int. J. Osteoarchaeol..

[81] Bronk Ramsey C (1995). Radiocarbon calibration and analysis of stratigraphy: The OxCal program. Radiocarbon.

[82] Bronk Ramsey C, Dee M, Lee S, Nakagawa T, Staff R (2010). Developments in the calibration and modeling of radiocarbon dates. Radiocarbon.

[83] Ferreira JP, Leitão I, Santos-Reis M, Revilla E (2011). Human-related factors regulate the spatial ecology of domestic cats in sensitive areas for conservation. PLoS ONE.

[84] Pirie TJ, Thomas RL, Fellowes MDE (2022). Pet cats (*Felis catus*) from urban boundaries use different habitats, have larger home ranges and kill more prey than cats from the suburbs. Landsc. Urban Plan..

[85] Bocherens H (1997). Paleobiological implications of the isotopic signatures (^13^C, ^15^N) of fossil mammal collagen in Scladina cave (Sclayn, Belgium). Quat. Res..

[86] Longin R (1971). New method of collagen extraction for radiocarbon dating. Nature.

[87] Boudin M, Boeckx P, Vandenabeele P, Van Strydonck M (2013). Improved radiocarbon dating of contaminated protein-containing archaeological samples via cross-flow nanofiltrated amino acids. Rapid Commun. Mass Spectrom..

[88] Wojcieszak M, Van Den Brande T, Ligovich G, Boudin M (2020). Pretreatment protocols performed at the Royal Institute for Cultural Heritage (RICH) prior to AMS ^14^C measurements. Radiocarbon.

[89] Hammer Ø (2021). PAST. PAleontological Statistics. Version 4.05 Reference manual.

[90] Hammer Ø, Harper DAT, Ryan PD (2001). PAST: Paleontological statistics software package for education and data analysis. Palaeontol. Electron..

[91] Rohland N, Glocke I, Aximu-Petri A, Meyer M (2018). Extraction of highly degraded DNA from ancient bones, teeth and sediments for high-throughput sequencing. Nat. Protoc..

[92] Nguyen LT, Schmidt HA, Von Haeseler A, Minh BQ (2015). IQ-TREE: A fast and effective stochastic algorithm for estimating maximum-likelihood phylogenies. Mol. Biol. Evol..

