# Supplementary material for: Stable isotopes unveil one millennium of domestic cat paleoecology in Europe
Source: Sci Rep. 2022 Jul 27;12:12775. doi: 10.1038/s41598-022-16969-8 (PMC9329303; doi:10.1038/s41598-022-16969-8)
Supplement: Supplementary file 1 — Supplementary Information 1. [file 41598_2022_16969_MOESM1_ESM.pdf]

Supplementary Materials for

Stable isotopes unveil one millennium of domestic cat paleoecology in Europe

Magdalena Krajcarz*, Wim Van Neer, Maciej T. Krajcarz, Danijela Popović, Mateusz Baca, Bea De Cupere, Quentin Goffette, Hans Christian Küchelmann, Anna Gręzak, Urszula Iwaszczuk, Claudio Ottoni, Katrien Van de Vijver, Jarosław Wilczyński, Anna Mulczyk, Jan Wiejacki, Daniel Makowiecki, Hervé Bocherens

*Corresponding author. Email: magkrajcarz@umk.pl

This PDF file includes:

Supplementary text
Figs. S1 to S3
Tables S1 to S16

Other Supplementary Materials for this manuscript include the following:

Data S1 to S8

SUPPLEMENTARY TEXT

Quality assurance and control data in isotopic measurements

1. *Analysis in the Stable Isotopes Laboratory at the Institute of Geological Sciences, Polish Academy of Sciences (Warszawa, Poland)*

The lab used two internal standards, a powdered rib bone of gray seal and a powdered tibia of European elk from the osteological collection of the Institute of Archaeology, Nicolaus Copernicus University in Toruń (Poland) (Table S16). Samples of both these internal standards were proceeded with each run of collagen extraction (typically, both internal standards per each 18 samples) and measured along the samples to control the repeatability of the collagen extraction and of the stable isotope measurements.

Stable isotopic composition of nitrogen and carbon was determined using a Thermo Flash EA 1112HT elemental analyzer connected to a Thermo Delta V Advantage isotope ratio mass spectrometer (IRMS) in a continuous flow system (Thermo Scientific). Minimal weight of samples depended on the wt% amount of both elements. Samples were wrapped in tin capsules and combusted at 1020°C. Released gases (CO_2 and N_2) were purified on a water trap, separated in a GC column and transferred to MS source through a capillary. Isotope ratios were reported as delta (δ) notation and expressed relative to VPDB for $\delta^{13}C$ and to atmospheric nitrogen (AIR) for $\delta^{15}N$. Delta values were normalized to calibration curves. Three-point calibration curves for $\delta^{13}C$ and $\delta^{15}N$ were established each analytical day, based on international standards USGS40, USGS41 and IAEA-600 (Table S16). The repeatability of isotopic measurements (the 1σ standard deviation from many years for the measurements of the same standard with the device) is 0.33 ‰VPDB for $\delta^{13}C$ and 0.43 ‰AIR for $\delta^{15}N$.

As the rule, a measurement of a sample was done twice (in two sub-samples) and the results were averaged, unless the difference between the sub-samples exceeded the expected repeatability; in such cases the measurements were repeated and if failed again, the collagen extraction was repeated.

2. *Analysis in the Division of Soil and Water Management of the KU Leuven (Leuven, Belgium)*

Stable isotopic composition of nitrogen and carbon was determined using a Thermo Flash HT/EA elemental analyzer linked to a Thermo Delta V Advantage isotope ratio mass spectrometer (IRMS) via a ConfloIV interface (Thermo Scientific). Minimal weight of samples depends on the wt% amount of both elements. Samples wrapped in tin capsules are combusted at 1020°C. Released gases (CO_2 and N_2) are separated on a GC column and transferred to MS source through a capillary. Isotope ratios are reported as delta (δ) values and expressed relative to VPDB for $\delta^{13}C$ and to atmospheric nitrogen (AIR) for $\delta^{15}N$. Three-point calibration curves for $\delta^{13}C$ and $\delta^{15}N$ used a combination of an international standard IAEA-600 and two in-house standards (Leucine and muscle tissue of Pacific Tuna), which were previously calibrated versus certified standards (Table S16). Three-four replicates of each of these standards were measured along the collagen samples to calibrate the data and to quantify the repeatability of the stable isotope measurements. The repeatability of isotopic measurements (the 1σ standard deviation of standards) was 0.15 ‰ for both $\delta^{13}C$ and $\delta^{15}N$.

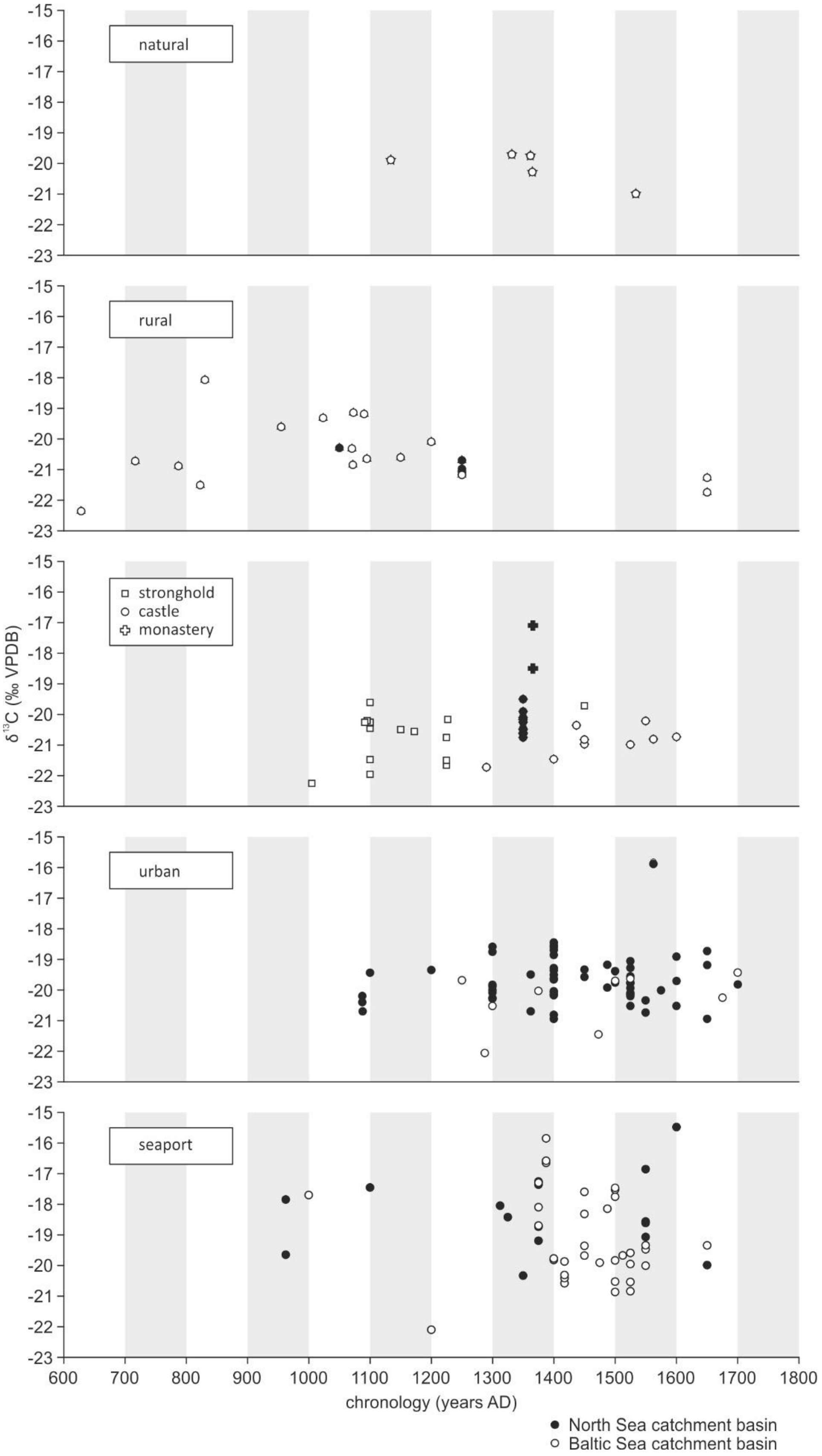

Fig. S1. $\delta^{13}C$ values in cats vs. specimen chronology, arranged by site socio-economic context. Presented chronology for each specimen is the central point (±1-year accuracy) of the site chronology range or the central point of the 95.4% probability range of the radiocarbon date. For full chronology ranges see Dataset S1 and S2.

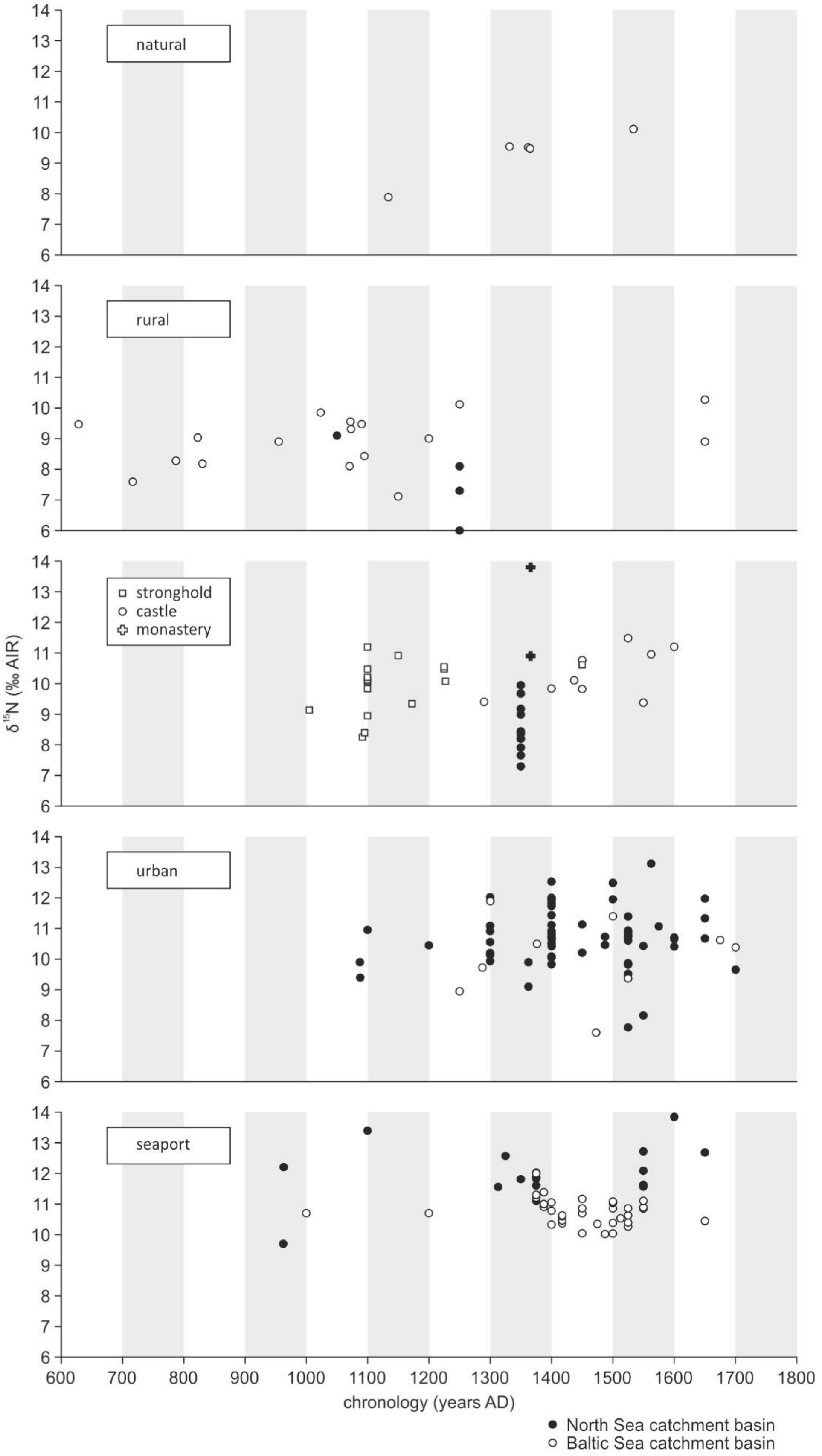

Fig. S2. $\delta^{15}N$ values in cats vs. specimen chronology, arranged by site socio-economic context. Presented chronology for each specimen is the central point (±1-year accuracy) of the site chronology range or the central point of the 95.4% probability range of the radiocarbon date. For full chronology ranges see Dataset S1 and S2.

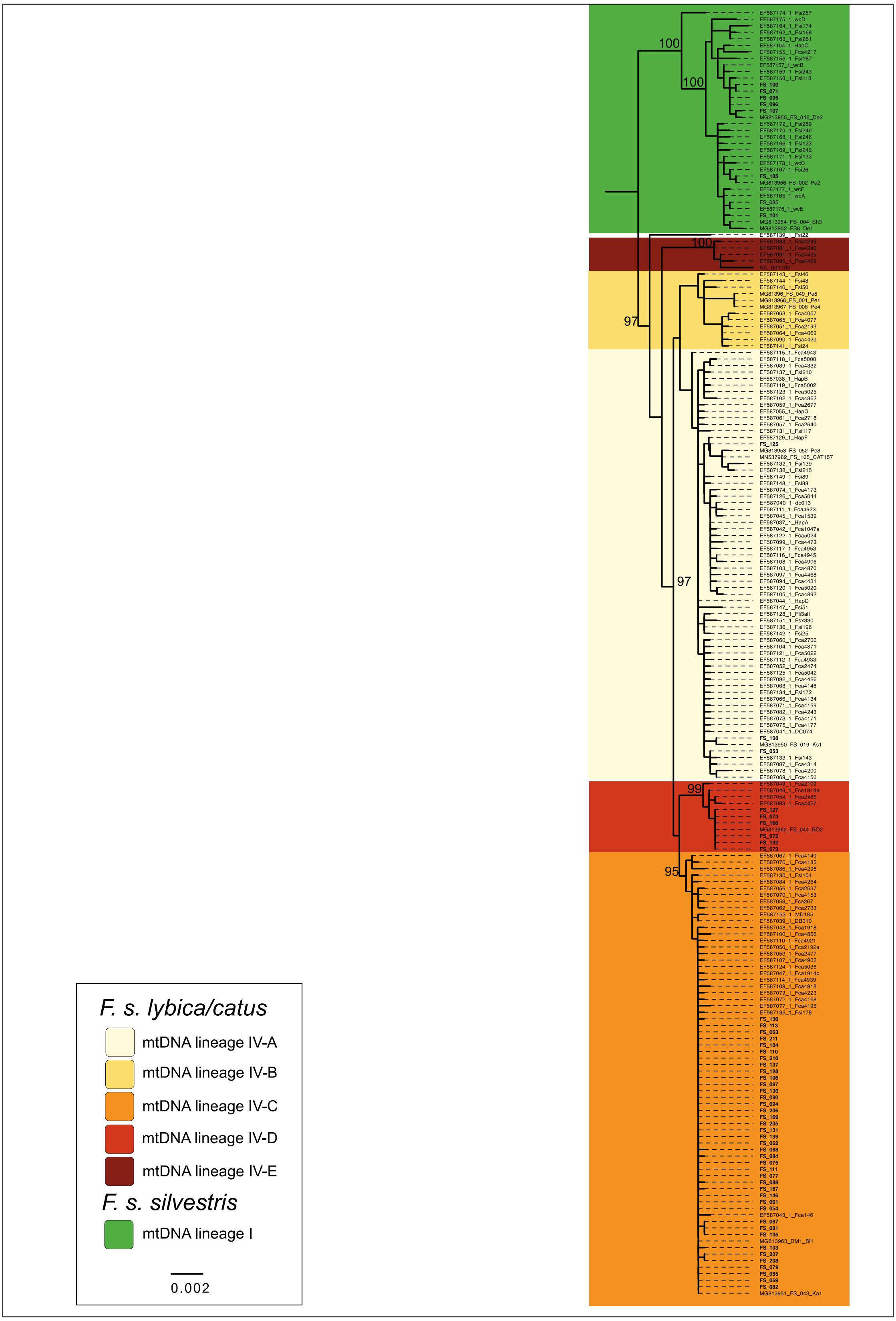

Fig. S3. The Maximum Likelihood Tree based on 2,604 bp of mtDNA. Sequences obtained in this study are shown in bold font. Numbers at the nodes represent bootstrap supports greater than 90 for the main mtDNA lineages.

Table S1. **Summary statistics of δ^{13}C and δ^{15}N values in cats (all domestic cats and unidentified) grouped by chronology**

	N	Min	Max	Mean	Median	Std. error	Stand. dev	Variance
all, δ^{13}C	188	-22.4	-15.5	-19.76596	-19.9	0.08841503	1.212286	1.469637
pre-AD 1250 (AD 1250 incl.), δ^{13}C	47	-22.4	-17.5	-20.3149	-20.3	0.162257	1.112377	1.237382
post-AD 1250, δ^{13}C	141	-22.1	-15.5	-19.583	-19.8	0.100409	1.192294	1.421565
all, δ^{15}N	188	6.0	13.8	10.34947	10.5	0.09530895	1.306811	1.707754
pre-AD 1250 (AD 1250 incl.), δ^{15}N	47	6.0	13.4	9.470213	9.5	0.194464	1.333175	1.777354
post-AD 1250, δ^{15}N	141	7.3	13.8	10.64255	10.7	0.097887	1.16234	1.351033

Table S2. Analysis of variance in δ^{13}C between pre-AD 1250 (AD 1250 included) and post-AD 1250 cats

Kruskal-Wallis test for equal medians

sea catchment area	*p* (same):
both	0.0001405
Baltic Sea	0.01531
North Sea	0.1177

$p<0.05$: there is a significant difference between sample medians

$p>0.05$: there is not any significant difference between sample medians

Table S3. Analysis of variance in $\delta^{15}N$ between pre-AD 1250 (AD 1250 included) and post-AD 1250 cats

Kruskal-Wallis test for equal medians

sea catchment area	*p* (same):
both	3.15E-08
Baltic Sea	1.187E-06
North Sea	0.03134

$p<0.05$: there is a significant difference between sample medians

$p>0.05$: there is not any significant difference between sample medians

SUMMARY STATISTICS

Table S4. Summary statistics of δ^{13}C values in cats (all domestic cats and unidentified) in the Baltic Sea catchment basin grouped by site socio-economic context, site geomorphology, distance from the sea, and chronology

	N	Min	Max	Mean	Median	Std. error	Stand. dev	Variance
site socio-economic context								
stronghold	17	-22.3	-19.6	-20.7235	-20.5	0.191459	0.789406	0.623162
urban	9	-22.1	-19.3	-20.2778	-20	0.319191	0.957572	0.916944
seaport	36	-22.1	-15.9	-19.2139	-19.7	0.23122	1.387321	1.924659
rural	17	-22.4	-18.1	-20.4412	-20.7	0.266522	1.098897	1.207574
natural	5	-21	-19.7	-20.14	-19.9	0.237908	0.531977	0.283
castle	9	-21.7	-20.2	-20.9	-20.8	0.15899	0.47697	0.2275
site geomorphology								
inland	19	-21.5	-18.1	-20.0158	-20.2	0.180036	0.78476	0.615848
lake shore	4	-21.5	-20.4	-20.925	-20.9	0.228674	0.457347	0.209167
river valley	21	-22.3	-19.3	-20.7238	-20.7	0.179139	0.820917	0.673905
sea coast	35	-20.9	-15.9	-19.1314	-19.7	0.222279	1.31502	1.729277
lagoon/fiord	14	-22.4	-19.6	-21	-21.05	0.242015	0.905539	0.82
distance from the sea								
0-100 km	65	-22.4	-15.9	-19.9477	-20.3	0.174823	1.409467	1.986596
100-200 km	11	-21.2	-18.1	-20.2636	-20.5	0.265355	0.880083	0.774546
200-300 km	8	-22.3	-19.7	-20.675	-20.25	0.345248	0.97651	0.953571
400-500 km	9	-21	-19.1	-19.7667	-19.7	0.198606	0.595819	0.355
chronology								
pre-AD 1250 (AD 1250 incl.)	35	-22.4	-17.7	-20.4714	-20.5	0.180688	1.068966	1.142689
post-AD 1250	58	-22.1	-15.9	-19.7638	-19.85	0.173584	1.321973	1.747613

Table S5. **Summary statistics of δ^{15}N values in cats (all domestic cats and unidentified) in the Baltic Sea catchment basin grouped by site socio-economic context, site geomorphology, distance from the sea, and chronology**

	N	Min	Max	Mean	Median	Std. error	Stand. dev	Variance
site socio-economic context								
stronghold	17	8.3	11.2	9.941176	10.1	0.2067	0.852246	0.726324
urban	9	7.6	11.9	10.04444	10.4	0.43688	1.31064	1.717778
seaport	36	10	12	10.74722	10.75	0.070989	0.425935	0.181421
rural	17	7.1	10.3	8.923529	9	0.213949	0.882135	0.778162
natural	5	7.9	10.1	9.3	9.5	0.368782	0.824621	0.68
castle	9	9.4	11.5	10.33333	10.1	0.267187	0.801561	0.6425
site geomorphology								
inland	19	7.6	11.9	9.157895	9.3	0.253319	1.104192	1.21924
lake shore	4	9	11.5	10.4	10.55	0.549242	1.098484	1.206667
river valley	21	7.1	11.4	9.709524	9.7	0.229067	1.049717	1.101905
sea coast	35	10	12	10.74857	10.8	0.073034	0.432075	0.186689
lagoon/fiord	14	8.9	11.2	10.12857	10.25	0.176482	0.660336	0.436044
distance from the sea								
0-100 km	65	7.6	12	10.41846	10.5	0.098861	0.797044	0.635279
100-200 km	11	7.6	11	9.363636	9.6	0.348052	1.154359	1.332545
200-300 km	8	7.1	11.4	9.2125	9.25	0.483454	1.367414	1.869821
400-500 km	9	7.9	10.1	9.288889	9.5	0.20169	0.605071	0.366111
chronology								
pre-AD 1250 (AD 1250 incl.)	35	7.1	11.2	9.402857	9.5	0.17463	1.033124	1.067345
post-AD 1250	58	7.6	12	10.48966	10.6	0.099414	0.757116	0.573224

Table S6. **Summary statistics of $\delta^{13}C$ values in cats (all domestic cats and unidentified) in the North Sea catchment basin grouped by site socio-economic context, site geomorphology, distance from the sea, and chronology**

	N	Min	Max	Mean	Median	Std. error	Stand. dev	Variance
site socio-economic context								
castle	11	-20.8	-19.5	-20.3	-20.3	0.111192	0.368782	0.136
urban	61	-20.9	-15.8	-19.6853	-19.8	0.101863	0.795579	0.632945
seaport	17	-20.3	-15.5	-18.2588	-18.4	0.298779	1.231898	1.517574
rural	4	-21.1	-20.3	-20.775	-20.85	0.179699	0.359398	0.129167
monastery	2	-18.5	-17.1	-17.8	-17.8	0.7	0.98995	0.98
site geomorphology								
inland	16	-21.1	-19.5	-20.3875	-20.4	0.107578	0.43031	0.185167
river valley	67	-20.9	-15.5	-19.51045	-19.8	0.1247002	1.020715	1.041859
sea coast	12	-20.3	-17.1	-18.3167	-18.25	0.296912	1.028532	1.057879
distance from the sea								
0-100 km	82	-21.1	-15.5	-19.3878	-19.65	0.124182	1.124518	1.264541
100-200 km	12	-20.8	-19.5	-20.2583	-20.3	0.109723	0.380092	0.14447
200-300 km	1	-20.3	-20.3	-20.3	-20.3	0	0	0
chronology								
pre-AD 1250 (AD 1250 incl.)	12	-21.1	-17.5	-19.8583	-20.25	0.333816	1.156372	1.337197
post-AD 1250	83	-20.9	-15.5	-19.4566	-19.8	0.118879	1.083039	1.172974

Table S7. **Summary statistics of $\delta^{15}N$ values in cats (all domestic cats and unidentified) in the North Sea catchment basin grouped by site socio-economic context, site geomorphology, distance from the sea, and chronology**

	N	Min	Max	Mean	Median	Stand. error	Stand. dev.	Variance
site socio-economic context								
castle	11	7.3	9.9	8.545455	8.4	0.246932	0.818979	0.670727
urban	61	7.8	13.1	10.73443	10.7	0.126855	0.990772	0.981628
seaport	17	9.7	13.8	12.01176	11.9	0.222103	0.915753	0.838603
rural	4	6	9.1	7.625	7.7	0.654949	1.309898	1.715833
monastery	2	10.9	13.8	12.35	12.35	1.45	2.05061	4.205
site geomorphology								
inland	16	6	9.9	8.3875	8.35	0.259627	1.038509	1.0785
river valley	67	7.8	13.8	10.9403	10.8	0.1366568	1.118584	1.25123
sea coast	12	9.7	13.8	11.75	11.8	0.283512	0.982113	0.964546
distance from the sea								
0-100 km	82	6	13.8	10.91951	10.9	0.148641	1.345999	1.811713
100-200 km	12	7.3	9.9	8.641667	8.4	0.245091	0.849019	0.720833
200-300 km	1	9.1	9.1	9.1	9.1	0	0	0
chronology								
pre-AD 1250 (AD 1250 incl.)	12	6	13.4	9.666667	9.55	0.582879	2.019151	4.07697
post-AD 1250	83	7.3	13.8	10.7494	10.8	0.150505	1.371164	1.880091

SITE SOCIO-ECONOMIC CONTEXT: stronghold vs. urban vs. seaport vs. rural vs. natural vs. castle

Table S8. Analysis of variance in $\delta^{13}C$ between cats (all domestic cats an unidentified) grouped by site socio-economic context, Baltic Sea catchment basin

ANOVA/*Welch F*/Kruskal-Wallis (upper triangle: *p*(same); lower triangle: F; df or H; Hc)

	stronghold	urban	seaport	rural	natural	castle
stronghold	–	0.1361	*7.00E-06*	0.6913	0.1339	0.279
urban	2.197; 2.222	–	0.09641	0.7101	0.7737	0.1002
seaport	*25.29; 49.03*	2.755; 2.764	–	0.003023	0.1292	*5.34E-07*
rural	0.1569; 0.1576	0.1415; 1,24	8.78; 8.794	–	0.5648	*0.1526*
natural	2.216; 2.246	0.08647; 1,12	2.292; 2.302	0.3427; 1,20	–	0.01769
castle	1.162; 1.172	3.045; 1,16	*36.11; 38.38*	*2.186; 23.47*	7.547; 1,12	–

p<0.05: there is a significant difference between sample medians

p>0.05: there is not any significant difference between sample median

Table S9. **Analysis of variance in δ^{15}N between cats (all domestic cats an unidentified) grouped by site socio-economic context, Baltic Sea catchment basin**

ANOVA/*Welch F* (upper triangle: *p*(same); lower triangle: F; df)

	stronghold	urban	seaport	rural	natural	castle
stronghold	–	8.10E-01	*0.00147*	1.72E-03	0.1523	0.2662
urban	0.05938; 1,24	–	*0.1491*	0.0156	0.2767	0.5805
seaport	*13.6; 19.87*	*2.521; 8.427*	–	*1.13E-07*	2.06E-07	*0.168*
rural	11.7; 1,32	6.775; 1,24	*65.45; 19.61*	–	0.4056	0.000534
natural	2.215; 1,20	1.298; 1,12	39.54; 1,39	0.7219; 1,20	–	0.041*
castle	1.296; 1,24	0.3182; 1,16	*2.241; 9.159*	15.96; 1,24	5.24; 1,12	–

p<0.05: there is a significant difference between sample medians

p>0.05: there is not any significant difference between sample medians

**p* is close to the 0.05 confidence level

Table S10. Analysis of variance in $\delta^{13}C$ between cats (all domestic cats an unidentified) grouped by site socio-economic context, North Sea catchment basin

ANOVA/*Welch F*/Kruskal-Wallis (upper triangle: *p*(same); lower triangle: F; df or H; Hc)

	castle	urban	seaport	rural	monastery
castle	–	0.002967	*2.94E-06*	4.49E-02*	*1.66E-01*
urban	8.797; 8.828	–	*0.00021*	0.003431	0.02158
seaport	*40.99; 20.12*	*20.42; 19.86*	–	8.14E-04	0.6211
rural	4.924; 1,13	8.531; 8.563	15.79; 1,19	–	*0.1287*
monastery	*12.44; 1.051*	5.26; 5.279	0.2535; 1,17	*16.95; 1.135*	–

p<0.05: there is a significant difference between sample medians

p>0.05: there is not any significant difference between sample medians

**p* is close to the 0.05 confidence level

Table S11. Analysis of variance in δ^{15}N between cats (all domestic cats an unidentified) grouped by site socio-economic context, North Sea catchment basin

ANOVA post hoc Tukey's test (Copenhaver-Holland 1988) (upper triangle: *p*(same); lower triangle: Tukey's Q)

	castle	urban	seaport	rural	monastery
castle	–	1.48E-08	4.36E-10	0.5071	2.80E-05
urban	9.537	–	8.97E-05	2.84E-07	0.1647
seaport	12.78	6.647	–	4.83E-10	0.9909
rural	2.25	8.598	11.27	–	3.39E-06
monastery	7.063	3.209	6.46E-01	7.787	–

$p<0.05$: there is a significant difference between sample medians

$p>0.05$: there is not any significant difference between sample medians

SITE GEOMORPHOLOGY: inland vs. lake shore vs. river valley vs. sea coast vs. lagoon/fiord

Table S12. Analysis of variance in δ^{13}C between cats (all domestic cats an unidentified) grouped by site geomorphology, Baltic Sea catchment basin

ANOVA/*Welch F*/Kruskal-Wallis (upper triangle: *p*(same); lower triangle: F; df or H; Hc)

	inland	lake shore	river valley	sea coast	lagoon/fiord
inland	–	0.0381	0.008379	*3.21E-03*	0.002215
lake shore	4.898; 1,21	–	0.6421	*0.00018*	*0.8261*
river valley	7.736; 1,38	0.2218; 1,23	–	*8.07E-07*	3.56E-01
sea coast	*9.559; 51.43*	*31.63; 10.52*	*31.11; 53.87*	–	4.35E-05
lagoon/fiord	11.13; 1,31	*0.05074; 10.46*	0.876; 1,33	16.67; 16.71	–

p<0.05: there is a significant difference between sample medians

p>0.05: there is not any significant difference between sample medians

Table S13. Analysis of variance in $\delta^{15}N$ between cats (all domestic cats an unidentified) grouped by site geomorphology, Baltic Sea catchment basin

ANOVA/*Welch F* (upper triangle: *p*(same); lower triangle: F; df)

	inland	lake shore	river valley	sea coast	lagoon/fiord
inland	–	5.35E-02*	0.1136	*5.44E-06*	6.47E-03
lake shore	4.187; 1,21	–	0.243	*0.5725*	0.5386
river valley	2.622; 1,38	1.436; 1,23	–	*2.31E-04*	0.1941
sea coast	*36.4; 21.04*	*0.3958; 3.107*	*18.68; 24.13*	–	3.27E-04
lagoon/fiord	8.526; 1,31	0.3948; 1,16	1.757; 1,33	15.04; 1,47	–

p<0.05: there is a significant difference between sample medians

p>0.05: there is not any significant difference between sample medians

**p* is close to the 0.05 confidence level

Table S14. **Analysis of variance in $\delta^{13}C$ between cats (all domestic cats an unidentified) grouped by site geomorphology, North Sea catchment basin**

ANOVA/ Kruskal-Wallis (upper triangle: *p*(same); lower triangle: F; df or H; Hc)

	inland	river valley	sea coast
inland	–	7.17E-05	9.82E-08
river valley	15.72; 15.77	–	0.000465
sea coast	53.04; 1,26	12.23; 12.25	–

$p<0.05$: there is a significant difference between sample medians

$p>0.05$: there is not any significant difference between sample medians

Table S15. **Analysis of variance in $\delta^{15}N$ between cats (all domestic cats an unidentified) grouped by site geomorphology, North Sea catchment basin**

ANOVA post hoc Tukey's test (Copenhaver-Holland 1988) (upper triangle: *p*(same); lower triangle: Tukey's Q)

	inland	river valley	sea coast
inland	–	4.84E-10	4.90E-10
river valley	11.9	–	5.16E-02*
sea coast	1.14E+01	3.35	–

$p<0.05$: there is a significant difference between sample medians

$p>0.05$: there is not any significant difference between sample medians

*p is close to the 0.05 confidence level

Table S16. Internal standards and calibration standards used in the isotopic analyses.

Standard	Compound	Used in the lab	$\delta^{13}C$	sd	$\delta^{15}N$	sd
Internal standard SEAL	collagen	W	-17.29	0.38	14.79	0.42
Internal standard ELK	collagen	W	-24.84	0.31	0.82	0.36
Internal standard Leucine	leucine	L	-13.47	0.07	0.92	0.06
Internal standard Tuna	Pacific Tuna muscle tissue	L	-18.72	0.09	13.27	0.07
USGS40	L-glutamic acid	W	-26.39*	0.04*	-4.52*	0.06*
USGS41	^{13}C- and ^{15}N-enriched L-glutamic acid	W	37.63*	0.05*	47.57*	*0.11
IAEA-600	caffeine	W, L	-27.771**	0.043**	1.0**	0.2**

W – Stable Isotope Lab, Institute of Geological Sciences, Polish Academy of Sciences, Warsaw (Poland)
L – Division of Soil and Water Management, KU Leuven, Leuven (Belgium)
* according to https://isotopes.usgs.gov
** according to https://nucleus.iaea.org
